# Supplementary material for: Foundational and Clinical Science Integration in a Team-Based Learning Module Modeling Care of a Patient With Dyslipidemia
Source: MedEdPORTAL. 2024 Apr 9;20:11397. doi: 10.15766/mep_2374-8265.11397 (PMC11001791; doi:10.15766/mep_2374-8265.11397)
Supplement: Supplementary file 1 — Preparation Resources.pptxReadiness Assurance Test.docxRAT Question Appeal Form.docxApplication Exercises.docxFacilitator Guide.docx [file mep_2374-8265.11397-s001.zip › _Educational Summary Report_11397.pdf]

MedEdPORTAL® The AAMC Journal of Teaching and Learning Resources

Original Publication Open Access

Foundational and Clinical Science Integration in a Team-Based Learning Module Modeling Care of a Patient With Dyslipidemia

Paul C. Megee, PhD*, Virginia Uhley, PhD, RDN, James Grogan, PhD, Alan Silverman, DO

*Corresponding author: megee@oakland.edu

Abstract

Introduction: Foundational and clinical science integration, a long-standing goal of undergraduate medical education, benefits learners by promoting retention of critical knowledge and skills as well as their transfer to the clinical setting. We implemented a team-based learning (TBL) module in which foundational knowledge and skills from the disciplines of biochemistry, nutrition, and genetics were leveraged in a simulated patient encounter for diagnosis and management of a patient with dyslipidemia. **Methods:** The TBL was deployed in a first-year medical student cardiovascular system course with 125 students over three academic years. Following individual and team readiness assurance tests (iRAT and tRAT, respectively), teams participated in an initial application exercise requiring consideration of clinical and laboratory data and other risk factors to engage the patient in a shared decision-making process. Using dietary and family history narratives in subsequent application exercises, teams completed recommendations for an individualized diet plan and an assessment of potential disease inheritance patterns to formulate appropriate patient care management strategies. **Results:** Student engagement with prelearning materials and session team activities was high as judged by RAT performance and application exercise outcomes: iRAT question performance ranged from 89% to 99% for individual items, and tRAT performance was routinely 100%. Learners reported that the exercises were impactful and believed the learned foundational knowledge and skills were transferable to future patient care. **Discussion:** The dyslipidemia TBL module provides an illustration for early clinical learners of how foundational knowledge and skills can be operationalized and transferred for optimal patient care.

Keywords
Dyslipidemia, Pedigree, Shared Decision-Making, Biochemistry & Cell Biology, Cardiovascular Medicine, Communication Skills, Genetics, Lifestyle Medicine, Nutrition, Team-Based Learning

Educational Objectives

By the end of this activity, learners will be able to:

1. Prioritize shared decision-making in patient consultations regarding risk factors for cardiovascular disease, including clinical and laboratory findings.
2. Evaluate dietary history and create an individualized dietary management plan that promotes cardiovascular risk reduction.
3. Construct and interpret a three-generation pedigree from family health history to assess potential genetic contributions to disease in a dyslipidemia patient.

Citation:
Megee PC, Uhley V, Grogan J, Silverman A. Foundational and clinical science integration in a team-based learning module modeling care of a patient with dyslipidemia. *MedEdPORTAL*. 2024;20:11397.
https://doi.org/10.15766/mep_2374-8265.11397

Introduction

Although underdiagnosed, familial hypercholesterolemia is one of the most commonly inherited metabolic diseases, occurring in approximately one in 250 individuals worldwide,[1,2] and can lead to significant cardiovascular disease risk and, in some cases, early death. Interventions that transfer[3] foundational science knowledge and skills in biochemistry, nutrition, and genetics are expected to have positive impacts in the care of dyslipidemia patients and are strongly recommended by health care professionals advocating for improved knowledge transfer of these disciplines to the clinic.[4-7] This team-based learning (TBL) resource is intended for first-year (M1) medical students and employs a simulated patient encounter to contextualize foundational science content and skills, providing a real-world, experiential example of how foundational knowledge and skills are required for effective clinical care of a dyslipidemia patient.[8] TBL, an effective pedagogical method used widely in undergraduate medical education to promote learning by

Copyright © 2024 Megee et al. This is an open-access publication distributed under the terms of the Creative Commons Attribution license.

fostering student engagement and collaboration,[9,10] can be used to teach the foundational sciences as support for informed patient care.

Several educational resources aligned with this exercise were identified, but none suited the aim of foundational science skill transfer for use in clinical practice. For example, a TBL simulating a cardiology patient encounter focuses on auscultation and heart sound discernment.[11] Similarly, resources that pertain to the inheritance of Mendelian (single-gene) disorders[12,13] are also available, but these emphasize formal inheritance risk calculation from supplied pedigrees.

Methods

The M1 preclinical curriculum at Oakland University William Beaumont (OUWB) School of Medicine began with foundational biomedical sciences and anatomy courses in an 18-week semester and four organ systems-based courses in a second semester of equal duration. Themes of hyperlipidemia, nutrition, and the pathogenesis of atherosclerosis were developed in the M1 cardiovascular system course. We presented the dyslipidemia TBL in week 5 of a 6-week cardiovascular system course, the third of four organ systems courses in the second semester of the M1 curriculum. Initially, we developed the TBL with the principal consideration that learners required integrated illustrations of knowledge and skills related to biochemistry, nutrition, and genetics disciplines to fully grasp their critical connections to clinical practice. We then used backward design[14] to devise preparatory materials and application exercises promoting experiential transfer[3] of knowledge and skills drawn from foundational science disciplines that would be crucial for care of a dyslipidemia patient.

Team Formation

We managed team assignments using a third-party application (e.g., GRumbler[15]) for creating teams whose members would have a diversity of traits, including sex, undergraduate major, and institution. Each 125-member class was divided into 21 teams consisting of five to seven students, a size that optimized individual participation.[16]

Description of Advance Preparation Resources

TBLs in the OUWB preclinical curriculum were typically designed to promote deeper comprehension and application of concepts presented previously within didactic sessions. Thus, advance preparation resources drew largely upon content presented in the cardiovascular system course and included selected portions of sessions entitled Biochemistry of Sterol Synthesis, Lipoprotein Particles and Cholesterol Transport, and Molecular Pathogenesis of Atherosclerosis (delivered in week 2); Pharmacology of Lipid Lowering Drugs (week 3); and Nutrition and Cardiovascular Diseases (week 4). Students also had to recall foundational concepts taught in the M1 biomedical sciences courses, including Mendelian and non-Mendelian patterns of inheritance and fatty acid and triglyceride synthesis. For student convenience and to emphasize key content, we consolidated selected PowerPoint slides from the aforementioned didactic sessions as advance preparatory materials (Appendix A). The advance preparatory materials also contained the top 10 take-home messages regarding clinical care recommendations for patients with elevated lipid levels as required reading.[17] We released session objectives and preparatory materials to students through the course learning management system 1 week prior to the scheduled TBL exercise.

Description of RAT Process and Immediate Feedback

Due to pandemic-imposed constraints on in-person instruction, we conducted TBL sessions remotely during the 2021 and 2022 courses, using videoconferencing for team and class discussions, and returned to in-person instruction in 2023. Sessions began with an individual readiness assurance test (iRAT; Appendix B), administered electronically using an optional web-based TBL software (e.g., InteDashboard [CognaLearn]), that measured students' understanding of the advance preparatory resources (Appendix A). Teams then retook the same assessment (if necessary, in videoconference team breakout rooms), with a team representative submitting team readiness assurance test (tRAT) answers using InteDashboard. This platform provided immediate feedback, alerting teams when an incorrect answer was submitted, thereby allowing teams to make new selections until the correct answer was chosen. Teams were awarded full credit for a correct answer chosen on the first attempt and progressively lost credit with subsequent answer attempts. RATs were completed without the use of class notes or other resources. Neither videoconferencing nor InteDashboard was essential for this TBL when conducted in-person.

Facilitators, three of whom were basic scientists with individual expertise in biochemistry, genetics, or nutrition and another who was a practicing cardiologist, reviewed RAT performance data as answers were submitted in real time in InteDashboard to identify potentially challenging concepts. Once the tRAT ended, facilitators addressed unclear concepts with the entire class. RAT question appeals could be submitted by teams, but not individual students, if they believed questions were ambiguously worded or tested concepts insufficiently explained in preparatory materials. Relevant information could be collected from a team on an appeals form (Appendix C) within 24 hours of completion of

Copyright © 2024 Megee et al. This is an open-access publication distributed under the terms of the Creative Commons Attribution license.

the TBL and then submitted through the TBL Oversight Team to TBL facilitators, who would review the submitted appeal after completion of the session and respond in a timely manner in writing. No appeals were received for this TBL.

Description of Team Application Activities

The application exercises (Appendix D) simulated activities intended to model clinical assessments performed by a primary care physician or cardiologist treating a patient with previously undiagnosed dyslipidemia. A simulated patient encounter helped learners bridge the gap between foundational science theory and clinical practice and generated more interest in the educational session than might have been achieved using other modalities.[18] These activities met the 4S criteria of TBL, including posing a significant problem administered to all teams, and each team had to submit its answers or work products (dietary plans and pedigrees) electronically to InteDashboard at the same time before in-depth discussion of submissions. A schema of the time allotted for RATs and each application exercise is presented in Table 1, and we have included a facilitator guide (Appendix E) with detailed considerations of the application exercises and criteria that may be used to evaluate team submissions.

The first exercise familiarized students with a patient presenting with chest pain and cardiovascular disease risk factors. Lipid panel data suggested an underlying dyslipidemia as a risk factor that might help elicit a cardiac cause of the patient's chest pain (only 20% of chest pain presentations in the primary care setting are related to underlying hypercholesterolemia, so this information was vital to direct attention to genetic and nutritional factors of dyslipidemias). Teams quickly identified a hyperlipidemia and were asked what was most critical to discuss with the patient. Given that many risk factors were modifiable, teams were challenged to consider, through discussion, the importance of shared decision-making in patient care. Incorrect options were all potential actions for a clinician based on special aspects of care but would not fit the criteria for being always necessary. The lipid data were helpful to guide subsequent application exercises on nutritional and genetic aspects of follow-up. Students had already demonstrated their understanding of statin treatment guidelines featured in prereading and assessed in RAT questions. The advance preparation materials also emphasized treatment guidance with pharmacotherapy, as well as the importance of shared decision-making, introducing students to this essential feature of patient care. Discussions of the data and their use as an indicator of cardiovascular disease risk, as well as the further consideration of all the patient's risk factors, were vital to suggesting the relevance of different types of information in the initial care of the patient (leading up to nutritional and genetic family history).

Table 1. TBL Facilitation Schema

TBL Activity	Assigned Time (111 Minutes)
Individual RAT	9 minutes (1.5 minutes/question)
Team RAT	12 minutes + 10 minutes for clarifications
Application exercise 1	10 minutes + 10 minutes for discussion
Application exercise 2	20 minutes + 10 minutes for discussion
Application exercise 3	20 minutes + 10 minutes for discussion

Abbreviations: RAT, readiness assurance test; TBL, team-based learning.

In the second application exercise, teams evaluated the supplied narrative regarding the patient's recent diet history, which teams should have recognized as a dietary pattern aligning well with a low-carbohydrate eating plan, and then submitted recommended changes to the dietary intake pattern that would promote a more heart-healthy diet. Submissions had to center on recommendations to increase fruit and vegetable intake, which were key components of the Therapeutic Lifestyle Change (TLC),[19] the Mediterranean diet,[20] and the Dietary Approaches to Stop Hypertension (DASH) dietary patterns.[21] Additional recommendations that aligned with a low-fat dietary intake pattern to reduce high cholesterol or a low-sodium diet to reduce high blood pressure were also expected. Following submission of dietary pattern recommendations, a facilitator chose several teams to present their interpretations of the patient's current diet and discuss the merits of their submitted recommendations.

In the third application exercise, teams had to interpret a supplied patient narrative regarding family health history to generate and interpret a three-generation family pedigree. Pedigree construction emphasized its utility as a tool in subsequent patient care decision-making.[6] The patient narrative was deliberately colloquial and lacked definitive diagnoses of family members' conditions to reflect that patients often lack full understanding or awareness of family members' relevant health information, especially that of older generations who might be deceased. Descriptions of some family members' conditions were designed to be open to interpretation regarding their relevance to potential cardiovascular conditions. Thus, we expected differing interpretations of patient narrative to be reflected appropriately in the submitted pedigrees, enhancing the value of the exercise. Following submission of pedigrees with interpretations of potential inheritance patterns, a number of teams were chosen to present how their pedigrees reflected interpretations of family health histories. Facilitator feedback was provided using several criteria, including correct organization and usage of widely accepted pedigree symbols and a determination

Copyright © 2024 Megee et al. This is an open-access publication distributed under the terms of the Creative Commons Attribution license.

by the team regarding whether the segregation pattern of relevant phenotypes was consistent with a particular pattern of inheritance.

Results

Approximately 375 M1 students have participated in the TBL over three academic years. Table 2 shows the percentage of correctly answered iRAT items by question in each course year, which ranges from approximately 89% to 99% on average over three course years. Team RAT scores were 100% for all questions over the three course years, indicating effective peer teaching in TBL teams. We attribute generally strong annual RAT performances to learners benefiting from succinct advance preparatory materials and being highly motivated to prepare for RATs. A primary consideration for students was that RAT items were assigned higher value than questions on course high-stakes exams. Our objective using the RAT assessments was to ensure that students would be prepared to meaningfully engage in team applications.

Learner satisfaction with the TBL session was gleaned from end-of-course evaluations, which indicated that learners believed the TBL module met the stated objectives. Of 117 respondents, 79% agreed or strongly agreed with the statement that "the knowledge and skills learned in the dyslipidemia TBL are transferable to future patient care," while 75% agreed or strongly agreed that "the dyslipidemia TBL integrated biochemistry, nutrition, and genetics content relevant to the care of patients with dyslipidemia." The following narrative statements provided in course evaluations addressed the perceived utility of the module. The facilitator "offered great insight from a clinician's perspective during the TBL to supplement our learning," and similarly, the facilitator "shared clinical wisdom during the TBL." Students also recognized the relevance of the exercises, with one stating that

> I am glad that we had a TBL involving this material. I think that learning about diet in a live discussion is a good way to learn about it rather than just memorizing the numbers. Any way to have additional live student discussions with this material would be helpful.

Other students stated that the facilitator "did a good job keeping her discussion within her scope of practice," "offered nice insight during our TBL discussion to supplement our learning," and "touched on some important dietary recommendations and was helpful during the TBL."

Table 2. iRAT Question Performance

	iRAT Performance (%) by Question Number					
**Year**	**1**	**2**	**3**	**4**	**5**	**6**
2021	100	97	92	98	98	99
2022	99	97	90	94	99	97
2023	98	94	86	93	97	90
Average	99	96	89	95	98	96

Abbreviation: iRAT, individual readiness assurance test.

Discussion

While better incorporation of the foundational sciences into clinical practice through transfer of acquired knowledge and skills is likely to enhance patient care, it has been challenging to achieve. Transfer is best promoted by strategies that clearly demonstrate the relevance of the foundational sciences to clinical practice.[22] The simulated dyslipidemia patient encounter TBL explicitly illustrates for preclinical medical students how foundational skills in nutrition assessment and genetic analysis can be deployed for effective patient care. Moreover, the exercise's focus on shared decision-making extends a curricular theme in preclinical, longitudinal courses that emphasizes the value of motivational interviewing in clinical practice.

The first application exercise outlines a case vignette that prompts students to quickly identify above-normal low-density lipoprotein cholesterol (LDL-C) levels, as well as additional factors that should be recognized by teams as indicative of high risk for atherosclerosis, suggesting the need to develop a patient care plan. Many of our teams were able to discern the seminal importance of a risk-reduction discussion with the patient, but it is helpful to review each option in the accompanying question (Appendix D) to orient students to the patient's most likely benefit from consideration of modifiable lifestyle factors. It is also helpful to point out to students that patients are most receptive to advice on modifiable risk factors when faced with a need for statin therapy. In team discussions, student teams clearly understood that the patient's LDL-C value indicated a dyslipidemia that would benefit from statin therapy and consideration of modifiable risks. Rarely, student teams considered that genetic history would also require discussion.

Teams were generally successful in their nutrition assessment of the patient's dietary history. Importantly, the teams were able to propose cogent recommendations based on the TLC, Mediterranean diet, and DASH dietary patterns. The most significant deficiency when present included the absence of a team's recognition of the ketogenic dietary pattern included in the patient's dietary history. Because this is a common dietary approach for many patients attempting weight loss, it is important that students recognize and understand it and its impact on

Copyright © 2024 Megee et al. This is an open-access publication distributed under the terms of the Creative Commons Attribution license.

a patient's health risks. It was not possible to simultaneously display and discuss each dietary recommendation submitted by all 21 teams during the allotted time, so a facilitator with medical nutrition therapy expertise emailed each team detailed written critiques in which the strengths and weaknesses of that team's dietary recommendations were summarized.

Teams were generally successful in interpreting the family health history from supplied narratives and used standard pedigree symbols to construct a three-generation pedigree that accurately recorded traits indicative of cardiovascular disorders related to a dyslipidemia. Importantly, teams used their pedigrees as diagnostic tools[23] to make a presumptive diagnosis of familial hypercholesterolemia in the index patient. Because it was not possible to simultaneously display and discuss each pedigree submitted by all 21 teams during the allotted time, a facilitator with genetics expertise emailed each team detailed written critiques in which the strengths and weaknesses of that team's pedigree were summarized.

We anticipate that this TBL exercise could easily be modified to meet educational needs involving not only other foundational science disciplines but also medical ethics. For example, the genetics-related application exercise could be expanded to include a discussion of the potential implications of the diagnosis of an autosomal dominant trait, such as familial hypercholesterolemia, for first-degree relatives (parent, sibling, or child) of the index patient and to explore the counseling of patients regarding the ethical considerations surrounding disclosure of such a diagnosis to family members. Similarly, the first application exercise could be expanded to include hyperlipidemia classification, based upon the lipid data provided, to assist learners in the use of a classification rubric. The Dutch Lipid Clinic Network criteria[24] are aligned with the practice guidelines at our affiliated health care system (Corewell Health), where students will rotate in year 3. Individualized classification using polygenic associations is also possible using the Dutch Lipid Clinic Network criteria and available evidence.[25] Moreover, this TBL would lend itself well to the addition of an application exercise exploring the pharmacology of contemporary dyslipidemia treatment.

In the earliest iterations of this TBL, team members were asked to simulate the dyslipidemia patient and were provided with scripted dietary and family health history narratives. Other team members, in separate application exercises, interviewed the simulated patient to elicit dietary and family histories, respectively, in response to appropriately posed questions. We provided learners with a review article that described how to appropriately elicit genetic family histories as preparatory material. The TBL subsequently underwent revisions, driven by both facilitator and student evaluation. As a result, we discontinued the interviewing component due to the facilitators' inability to provide immediate feedback to all teams regarding interviewing skills.[26] This decision was coincident with the SARS-CoV-2 pandemic, which would have created further barriers for the provision of effective feedback regarding patient interviewing skills.

Initial student feedback revealed that some learners questioned the relevance of the dietary evaluation and pedigree building/interpretation exercises to patient care, believing that these activities would be more appropriately performed by other health care professionals. We addressed this perception by adding a practicing cardiologist as a facilitator. While the clinician's inclusion did not result in TBL design or content revisions, the physician validated the utility and importance of these tools in the clinical diagnosis and management of cardiology patients, which reduced student concerns regarding relevance and demonstrated to learners the scientific underpinnings of clinical care.[27,28] This observation supports previous findings that coteaching between basic scientists and clinicians models effective collaboration between disciplines and also reinforces for learners the importance of both foundational knowledge and skills in patient care.[29]

Education and training of medical students must continue to adapt to the changing needs of the 21st century health care delivery system. Educational activities that promote foundational science skill transfer to the clinical setting become even more crucial when considering competencies that future physicians may be asked to develop. For example, the proliferation of direct-to-consumer genetic testing and the dearth of clinical geneticists increasingly place primary care physicians at the forefront of patient engagement for genetics-related issues. Furthermore, this trend toward personalized medicine is only expected to grow with increased foundational knowledge in disciplines such as nutrigenetics, which explores the interactions of genes and diet.

The iRAT performance was quite strong overall each year, raising a potential concern that learner engagement and peer teaching were impacted as a result. However, tRAT discussions have been routinely robust, and learner evaluations of the TBL module indicate that it was valued. Nevertheless, we have included additional RAT questions viewed by experienced facilitators as more challenging to further ensure robust team discussions (Appendix B). While our application exercises meet the simultaneous reporting requirement for TBL, simultaneous reveal using gallery walks was prohibitive due to the significant

Copyright © 2024 Megee et al. This is an open-access publication distributed under the terms of the Creative Commons Attribution license.

time required for learners to view and rate both dietary plan recommendations and pedigrees submitted by 20 other teams. However, the ability to view submissions in real time through InteDashboard provided facilitators with the ability to judiciously select submissions with strengths and weaknesses to enrich discussions following presentations by the relevant teams.

Appendices

A. Preparation Resources.pptx
B. Readiness Assurance Test.docx
C. RAT Question Appeal Form.docx
D. Application Exercises.docx
E. Facilitator Guide.docx

All appendices are peer reviewed as integral parts of the Original Publication.

Paul C. Megee, PhD: Associate Professor, Department of Foundational Medical Studies, Oakland University William Beaumont School of Medicine; ORCID: https://orcid.org/0000-0002-9397-2161

Virginia Uhley, PhD, RDN: Associate Professor, Department of Foundational Medical Studies, Oakland University William Beaumont School of Medicine

James Grogan, PhD: Professor, Department of Foundational Medical Studies, Oakland University William Beaumont School of Medicine

Alan Silverman, DO: Assistant Professor, Department of Internal Medicine, Oakland University William Beaumont School of Medicine; Clinical Assistant Professor, Department of Internal Medicine, Wayne State University School of Medicine

Disclosures
None to report.

Funding/Support
None to report.

Ethical Approval
Reported as not applicable.

References

1. Knowles JW, O'Brien EC, Greendale K, et al. Reducing the burden of disease and death from familial hypercholesterolemia: a call to action. *Am Heart J*. 2014;168(6):807-811. https://doi.org/10.1016/j.ahj.2014.09.001
2. Tokgozoglu L, Kayikcioglu M. Familial hypercholesterolemia: global burden and approaches. *Curr Cardiol Rep*. 2021;23(10): 151. https://doi.org/10.1007/s11886-021-01565-5
3. Norman G. Teaching basic science to optimize transfer. *Med Teach*. 2009;31(9):807-811. https://doi.org/10.1080/01421590903049814
4. Kris-Etherton PM, Akabas SR, Bales CW, et al. The need to advance nutrition education in the training of health care professionals and recommended research to evaluate implementation and effectiveness. *Am J Clin Nutr*. 2014;99(5): 1153S-1166S. https://doi.org/10.3945/ajcn.113.073502
5. Hyland K, Garber K, Dasgupta S. From helices to health: undergraduate medical education in genetics and genomics. *Per Med*. 2019;16(3):211-220. https://doi.org/10.2217/pme-2018-0081
6. Weiler T, Landa-Galindez A. Online interactive genetics education during internal medicine clinical clerkship. *Genet Med*. 2022; 24(6):1362-1371. https://doi.org/10.1016/j.gim.2022.02.015
7. Aspry KE, Van Horn L, Carson JAS, et al.; American Heart Association Nutrition Committee of the Council on Lifestyle and Cardiometabolic Health, Council on Cardiovascular and Stroke Nursing, Council on Cardiovascular Radiology and Intervention, and Stroke Council. Medical nutrition education, training, and competencies to advance guideline-based diet counseling by physicians: a science advisory from the American Heart Association. *Circulation*. 2018;137(23):e821-e841. https://doi.org/10.1161/CIR.0000000000000563
8. Lisk K, Agur AMR, Woods NN. Exploring cognitive integration of basic science and its effect on diagnostic reasoning in novices. *Perspect Med Educ*. 2016;5(3):147-153. https://doi.org/10.1007/S40037-016-0268-2
9. Fatmi M, Hartling L, Hillier T, Campbell S, Oswald AE. The effectiveness of team-based learning on learning outcomes in health professions education: BEME Guide no. 30. *Med Teach*. 2013;35(12):e1608-e1624. https://doi.org/10.3109/0142159X.2013.849802
10. Reimschisel T, Herring AL, Huang J, Minor TJ. A systematic review of the published literature on team-based learning in health professions education. *Med Teach*. 2017;39(12): 1227-1237. https://doi.org/10.1080/0142159X.2017.1340636
11. Jackson JM, Stacey RB, Korczyk SS, Williams DM. The simulated cardiology clinic: a standardized patient exercise supporting medical students' biomedical knowledge and clinical skills integration. *MedEdPORTAL*. 2020;16:11008. https://doi.org/10.15766/mep_2374-8265.11008
12. Thatcher J, Canfield P, Bauer L, Griffith BN. Pedigree analysis: a team-based learning activity. *MedEdPORTAL*. 2017;13:10557. https://doi.org/10.15766/mep_2374-8265.10557
13. Kerry J, Chisholm E. Self-directed active learning modules for pedigree analysis and genetic risk assessment. *MedEdPORTAL*. 2015;11:10172. https://doi.org/10.15766/mep_2374-8265.10172
14. Wiggins G, McTighe J. *Understanding by Design*. Association for Supervision and Curriculum Development; 1998.

Copyright © 2024 Megee et al. This is an open-access publication distributed under the terms of the Creative Commons Attribution license.

15. The GRumbler: introduction to the Group Rumbler (“GRumbler”). Harvard University: Malcolm K. Sparrow. Accessed February 27, 2024. https://scholar.harvard.edu/msparrow/grumbler
16. Haidet P, Levine RE, Parmelee DX, et al. Perspective: guidelines for reporting team-based learning activities in the medical and health sciences education literature. *Acad Med*. 2012;87(3): 292-299. https://doi.org/10.1097/ACM.0b013e318244759e
17. Grundy SM, Stone NJ, Bailey AL, et al. 2018 AHA/ACC/AACVPR/AAPA/ABC/ACPM/ADA/AGS/APhA/ASPC/NLA/PCNA guideline on the management of blood cholesterol: a report of the American College of Cardiology/American Heart Association Task Force on Clinical Practice Guidelines. *Circulation*. 2019;139(25): e1082-e1143. https://doi.org/10.1161/CIR.0000000000000625
18. Makransky G, Bonde MT, Wulff JSG, et al. Simulation based virtual learning environment in medical genetics counseling: an example of bridging the gap between theory and practice in medical education. *BMC Med Educ*. 2016;16:98. https://doi.org/10.1186/s12909-016-0620-6
19. National Cholesterol Education Program Expert Panel on Detection, Evaluation and Treatment of High Blood Cholesterol in Adults. Third report of the National Cholesterol Education Program (NCEP) Expert Panel on Detection, Evaluation, and Treatment of High Blood Cholesterol in Adults (Adult Treatment Panel III) final report. *Circulation.* 2002;106(25):3143-3421. https://doi.org/10.1161/circ.106.25.3143
20. Willett WC, Sacks F, Trichopoulou A, et al. Mediterranean diet pyramid: a cultural model for healthy eating. *Am J Clin Nutr*. 1995;61(6):1402S-1406S. https://doi.org/10.1093/ajcn/61.6.1402S
21. Appel LJ, Moore TJ, Obarzanek E, et al; DASH Collaborative Research Group. A clinical trial of the effects of dietary patterns on blood pressure. *N Engl J Med.* 1997;336(16):1117-1124. https://doi.org/10.1056/NEJM199704173361601
22. Malau-Aduli BS, Alele FO, Heggarty P, Teague PA, Sen Gupta T, Hays R. Perceived clinical relevance and retention of basic sciences across the medical education continuum. *Adv Physiol Educ*. 2019;43(3):293-299. https://doi.org/10.1152/advan.00012.2019
23. Mahon SM. The three-generation pedigree: a critical tool in cancer genetics care. *Oncol Nurs Forum*. 2016;43(5):655-660. https://doi.org/10.1188/16.ONF.655-660
24. Nordestgaard BG, Chapman MJ, Humphries SE, et al; European Atherosclerosis Society Consensus Panel. Familial hypercholesterolaemia is underdiagnosed and undertreated in the general population: guidance for clinicians to prevent coronary heart disease: consensus statement of the European Atherosclerosis Society. *Eur Heart J.* 2013;34(45):3478-3490. https://doi.org/10.1093/eurheartj/eht273
25. Trinder M, Li X, DeCastro ML, et al. Risk of premature atherosclerotic disease in patients with monogenic versus polygenic familial hypercholesterolemia. *J Am Coll Cardiol*. 2019;74(4):512-522. https://doi.org/10.1016/j.jacc.2019.05.043
26. Kurtz S, Silverman J, Benson J, Draper J. Marrying content and process in clinical method teaching: enhancing the Calgary–Cambridge guides. *Acad Med*. 2003;78(8):802-809. https://doi.org/10.1097/00001888-200308000-00011
27. Schmidt H. Integrating the teaching of basic sciences, clinical sciences, and biopsychosocial issues. *Acad Med*. 1998;73(9 suppl):S24-S31. https://doi.org/10.1097/00001888-199809001-00006
28. Willey JM, Lim YS, Kwiatkowski T. Modeling integration: co-teaching basic and clinical sciences medicine in the classroom. *Adv Med Educ Pract*. 2018;9:739-751. https://doi.org/10.2147/AMEP.S169740
29. Crow J, Smith L. Using co-teaching as a means of facilitating interprofessional collaboration in health and social care. *J Interprof Care*. 2003;17(1):45-55. https://doi.org/10.1080/1356182021000044139

Received: November 29, 2023
Accepted: January 14, 2024
Published: April 9, 2024

Copyright © 2024 Megee et al. This is an open-access publication distributed under the terms of the Creative Commons Attribution license.
