# Supplementary material for: Foundational and Clinical Science Integration in a Team-Based Learning Module Modeling Care of a Patient With Dyslipidemia
Source: MedEdPORTAL. 2024 Apr 9;20:11397. doi: 10.15766/mep_2374-8265.11397 (PMC11001791; doi:10.15766/mep_2374-8265.11397)
Supplement: Supplementary file 1 — Preparation Resources.pptxReadiness Assurance Test.docxRAT Question Appeal Form.docxApplication Exercises.docxFacilitator Guide.docx [file mep_2374-8265.11397-s001.zip › A. Advance Preparation Resources.pptx]

## Slide 1
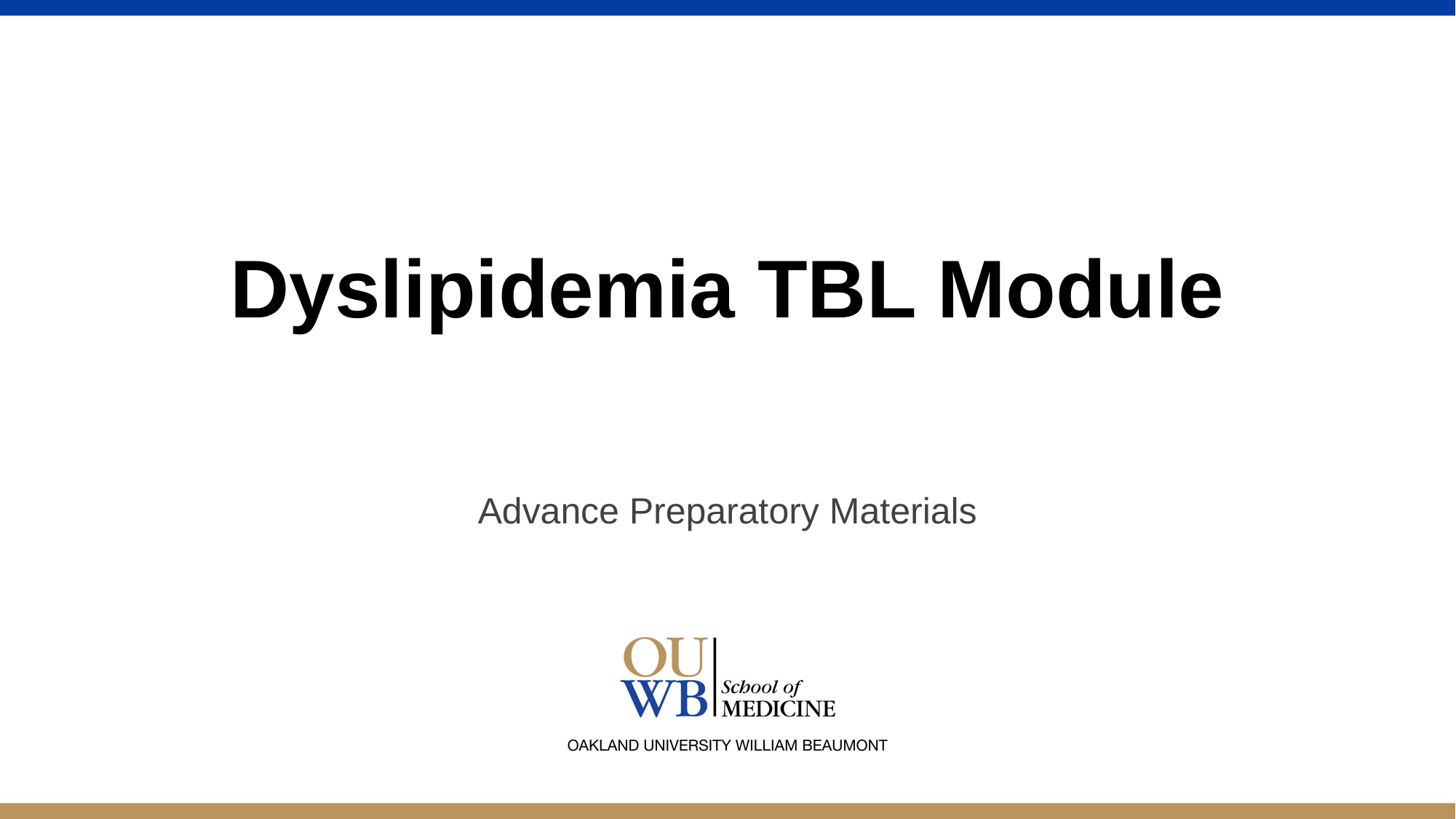

Dyslipidemia TBL Module
Advance Preparatory Materials

## Slide 2
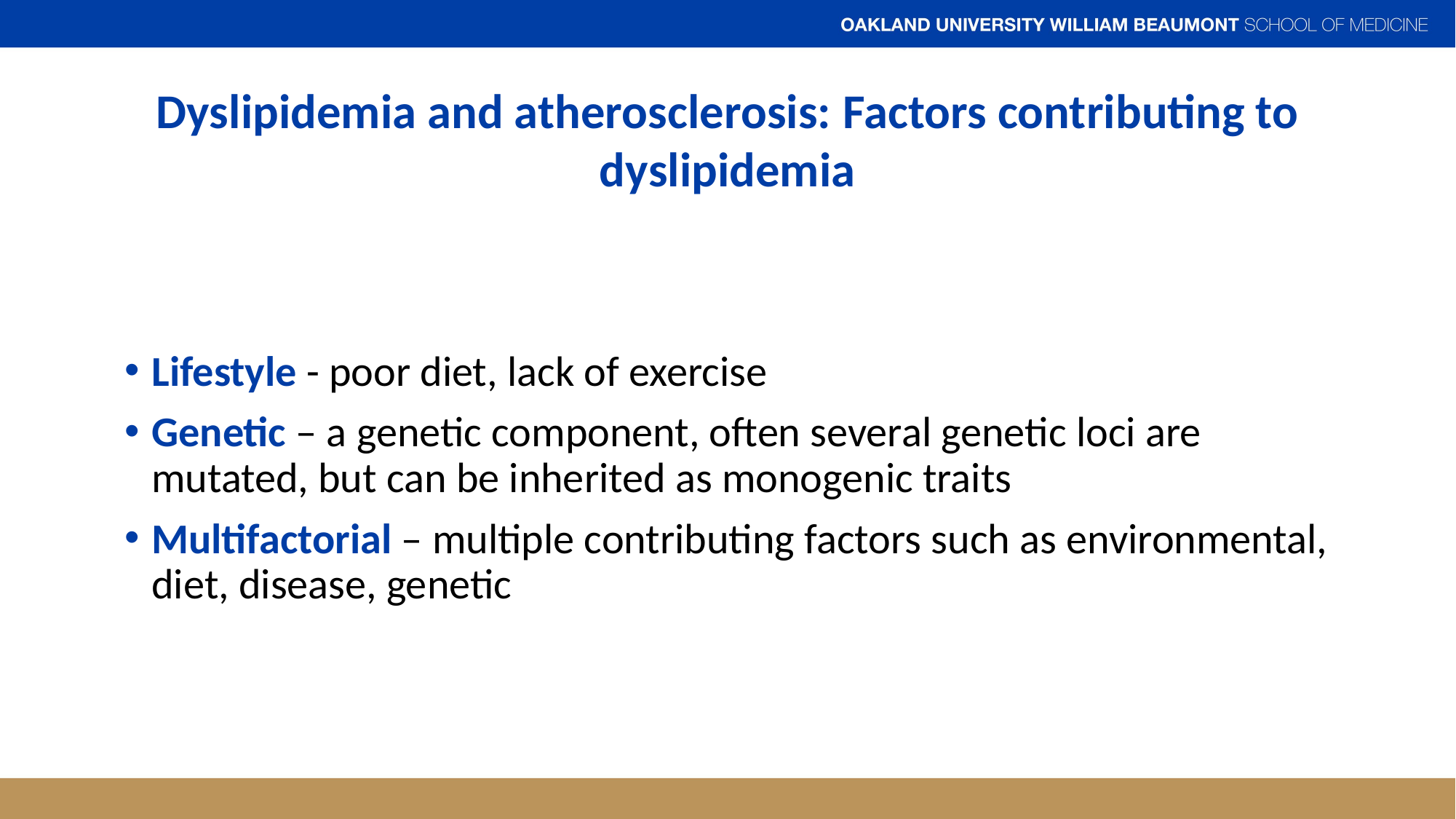

Dyslipidemia and atherosclerosis: Factors contributing to dyslipidemia
Lifestyle - poor diet, lack of exercise
Genetic – a genetic component, often several genetic loci are mutated, but can be inherited as monogenic traits
Multifactorial – multiple contributing factors such as environmental, diet, disease, genetic

## Slide 3
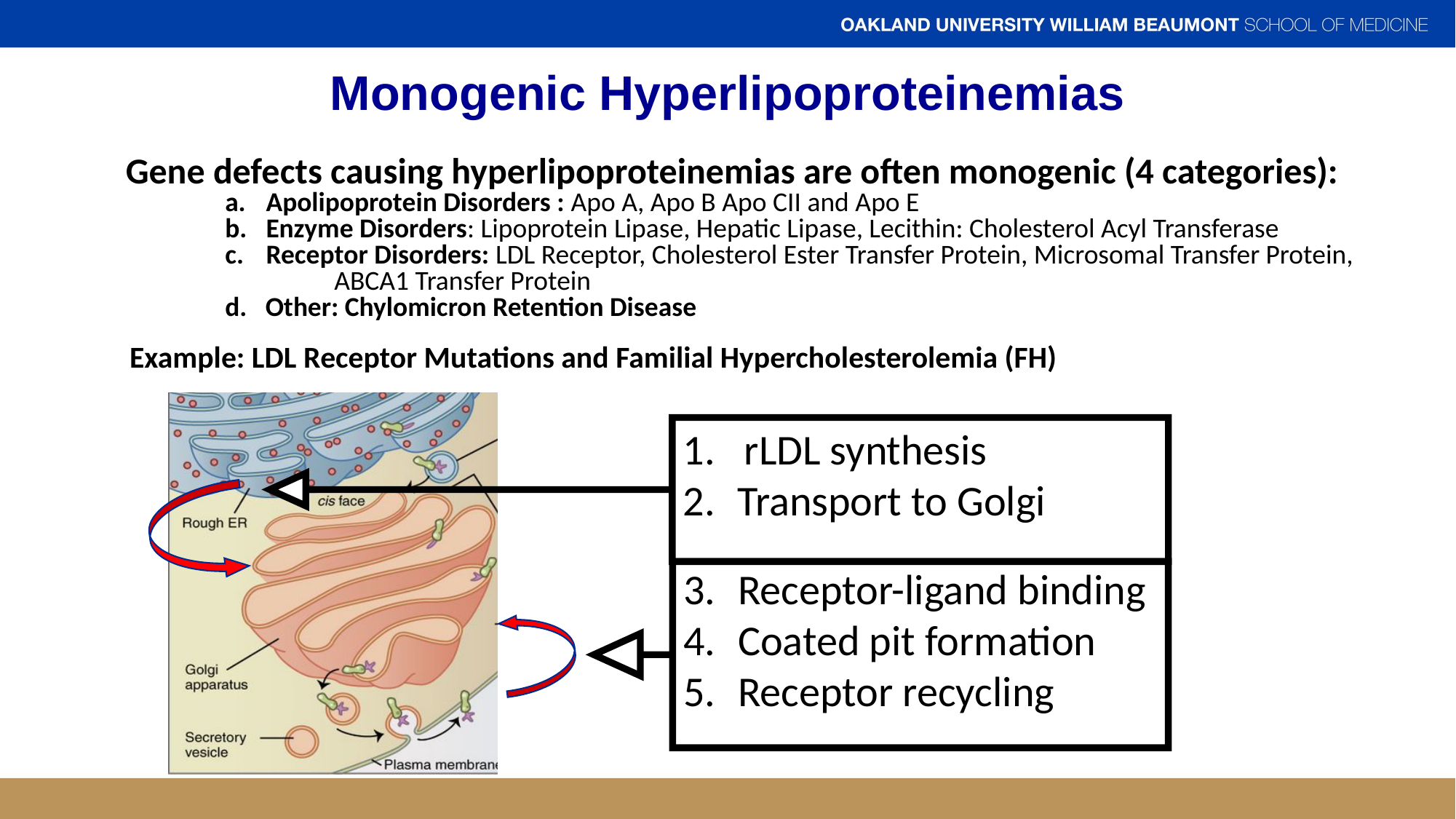

Monogenic Hyperlipoproteinemias
Gene defects causing hyperlipoproteinemias are often monogenic (4 categories):
Apolipoprotein Disorders : Apo A, Apo B Apo CII and Apo E
Enzyme Disorders: Lipoprotein Lipase, Hepatic Lipase, Lecithin: Cholesterol Acyl Transferase
Receptor Disorders: LDL Receptor, Cholesterol Ester Transfer Protein, Microsomal Transfer Protein,
	ABCA1 Transfer Protein
d. Other: Chylomicron Retention Disease
Example: LDL Receptor Mutations and Familial Hypercholesterolemia (FH)
rLDL synthesis
Transport to Golgi
Receptor-ligand binding
Coated pit formation
Receptor recycling

## Slide 4
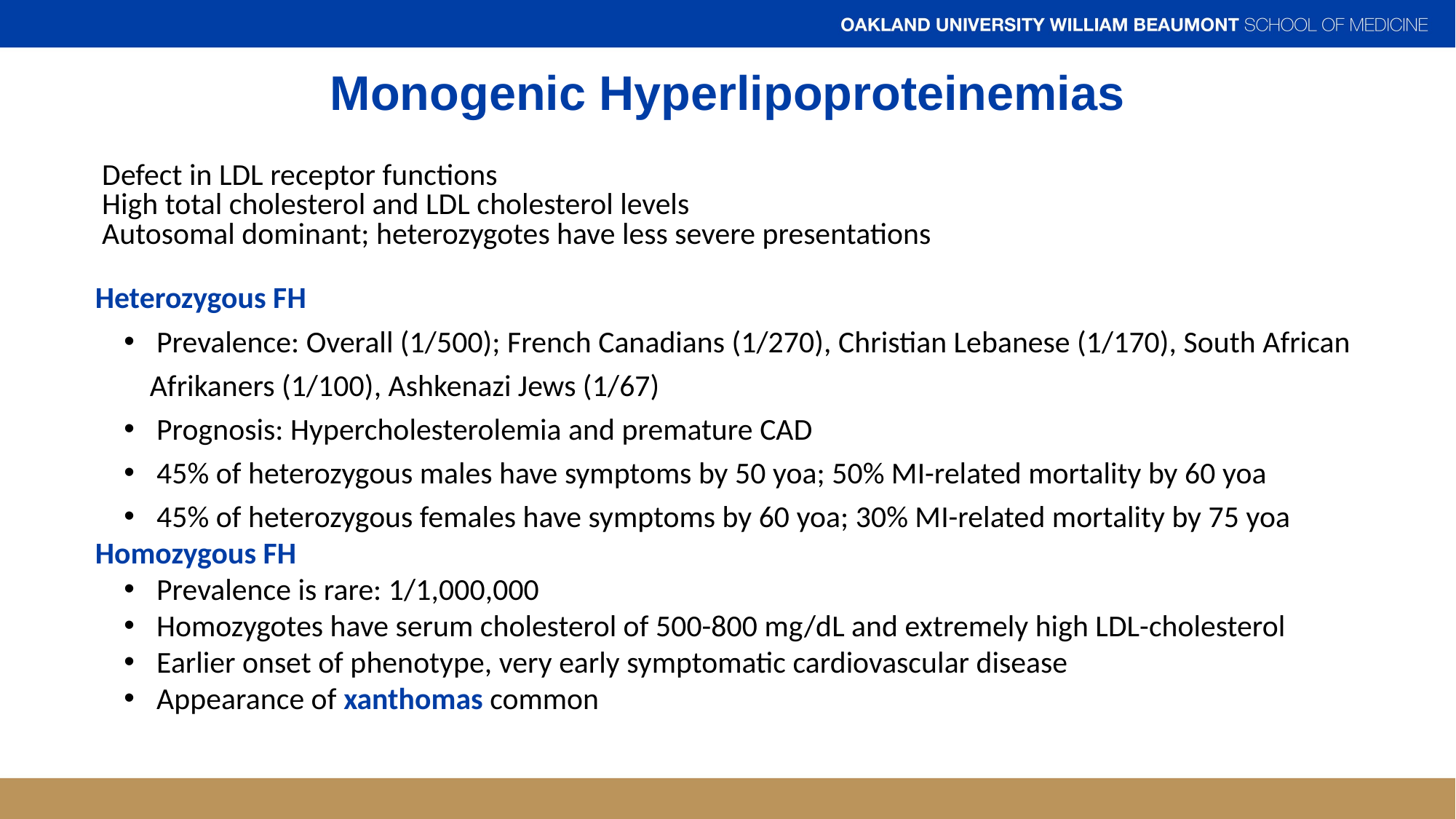

Monogenic Hyperlipoproteinemias
 Defect in LDL receptor functions
 High total cholesterol and LDL cholesterol levels
 Autosomal dominant; heterozygotes have less severe presentations
Heterozygous FH
 Prevalence: Overall (1/500); French Canadians (1/270), Christian Lebanese (1/170), South African Afrikaners (1/100), Ashkenazi Jews (1/67)
 Prognosis: Hypercholesterolemia and premature CAD
 45% of heterozygous males have symptoms by 50 yoa; 50% MI-related mortality by 60 yoa
 45% of heterozygous females have symptoms by 60 yoa; 30% MI-related mortality by 75 yoa
Homozygous FH
 Prevalence is rare: 1/1,000,000
 Homozygotes have serum cholesterol of 500-800 mg/dL and extremely high LDL-cholesterol
 Earlier onset of phenotype, very early symptomatic cardiovascular disease
 Appearance of xanthomas common

## Slide 5
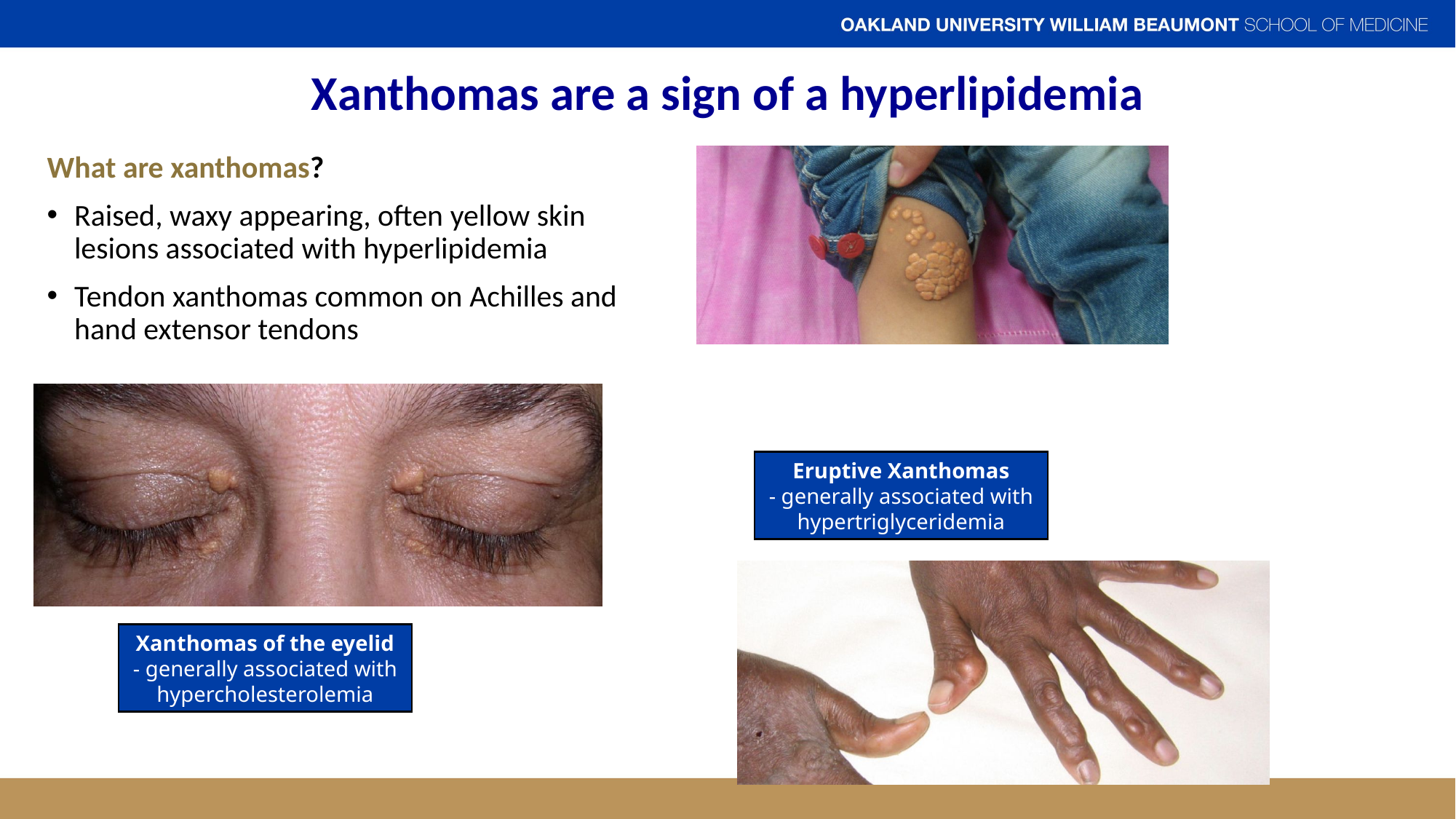

Xanthomas are a sign of a hyperlipidemia
What are xanthomas?
Raised, waxy appearing, often yellow skin lesions associated with hyperlipidemia
Tendon xanthomas common on Achilles and hand extensor tendons
Eruptive Xanthomas
- generally associated with
hypertriglyceridemia
Xanthomas of the eyelid
- generally associated with
hypercholesterolemia

## Slide 6
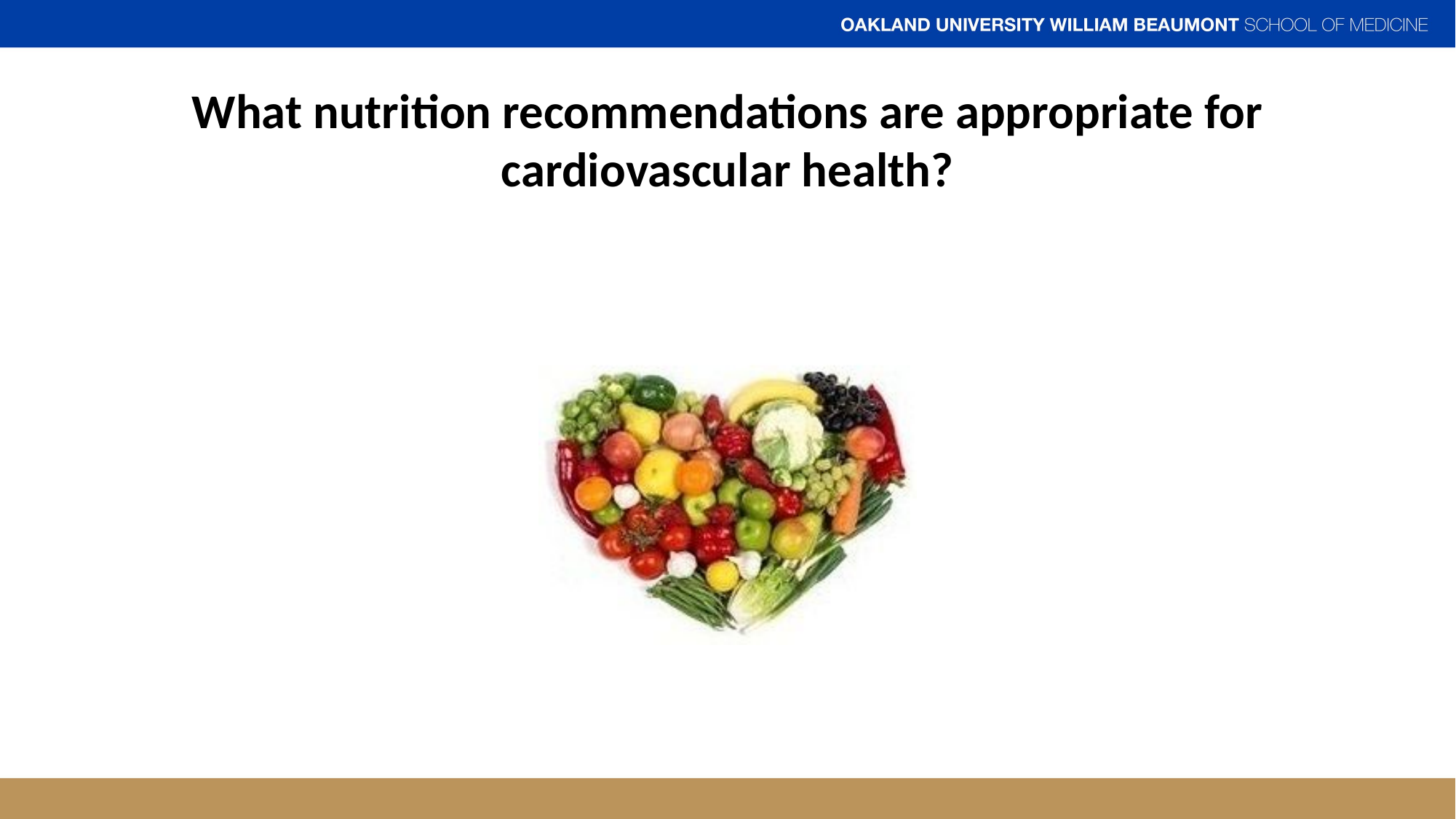

What nutrition recommendations are appropriate for cardiovascular health?

## Slide 7
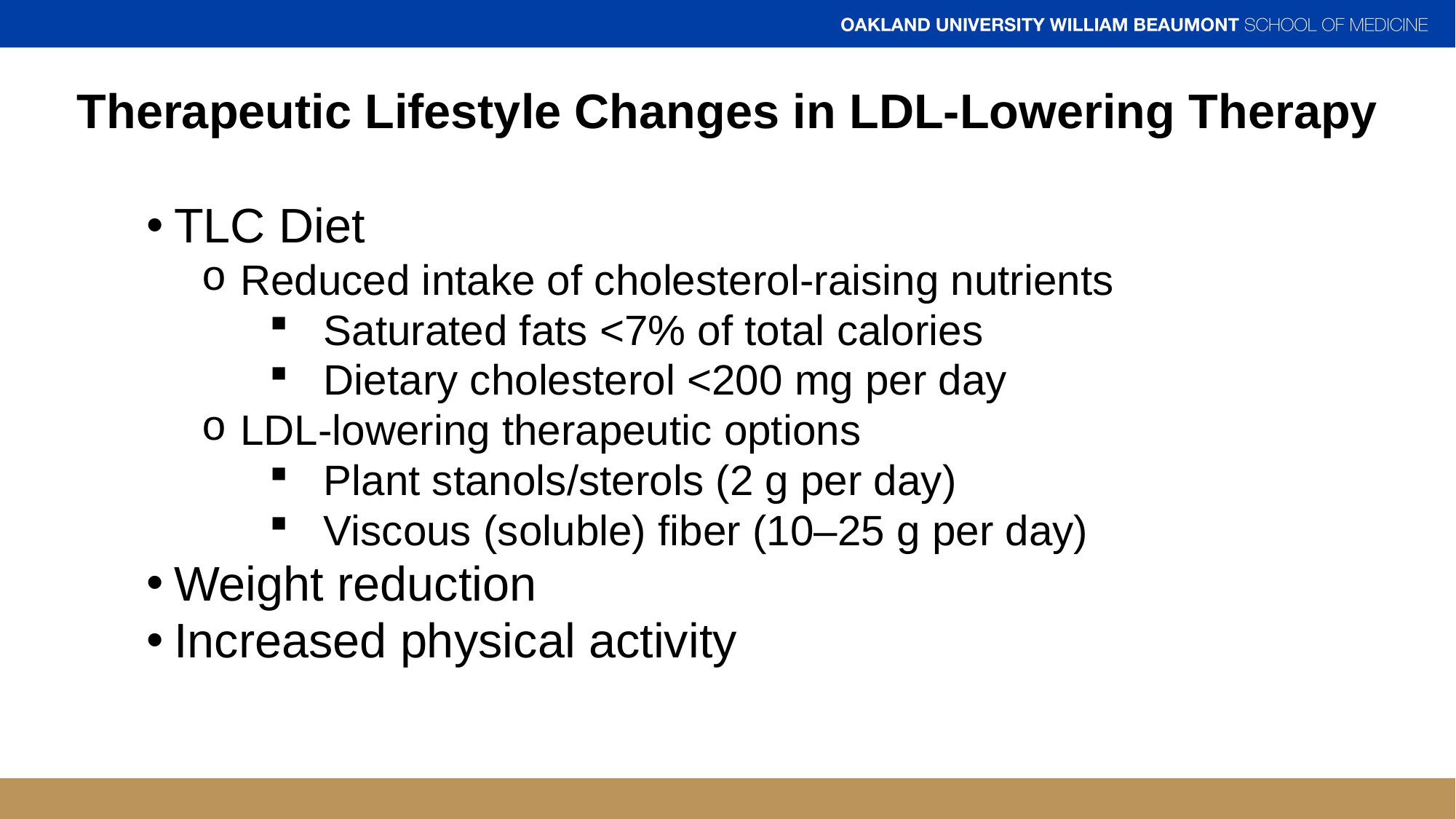

Therapeutic Lifestyle Changes in LDL-Lowering Therapy
TLC Diet
 Reduced intake of cholesterol-raising nutrients
Saturated fats <7% of total calories
Dietary cholesterol <200 mg per day
 LDL-lowering therapeutic options
Plant stanols/sterols (2 g per day)
Viscous (soluble) fiber (10–25 g per day)
Weight reduction
Increased physical activity

## Slide 8
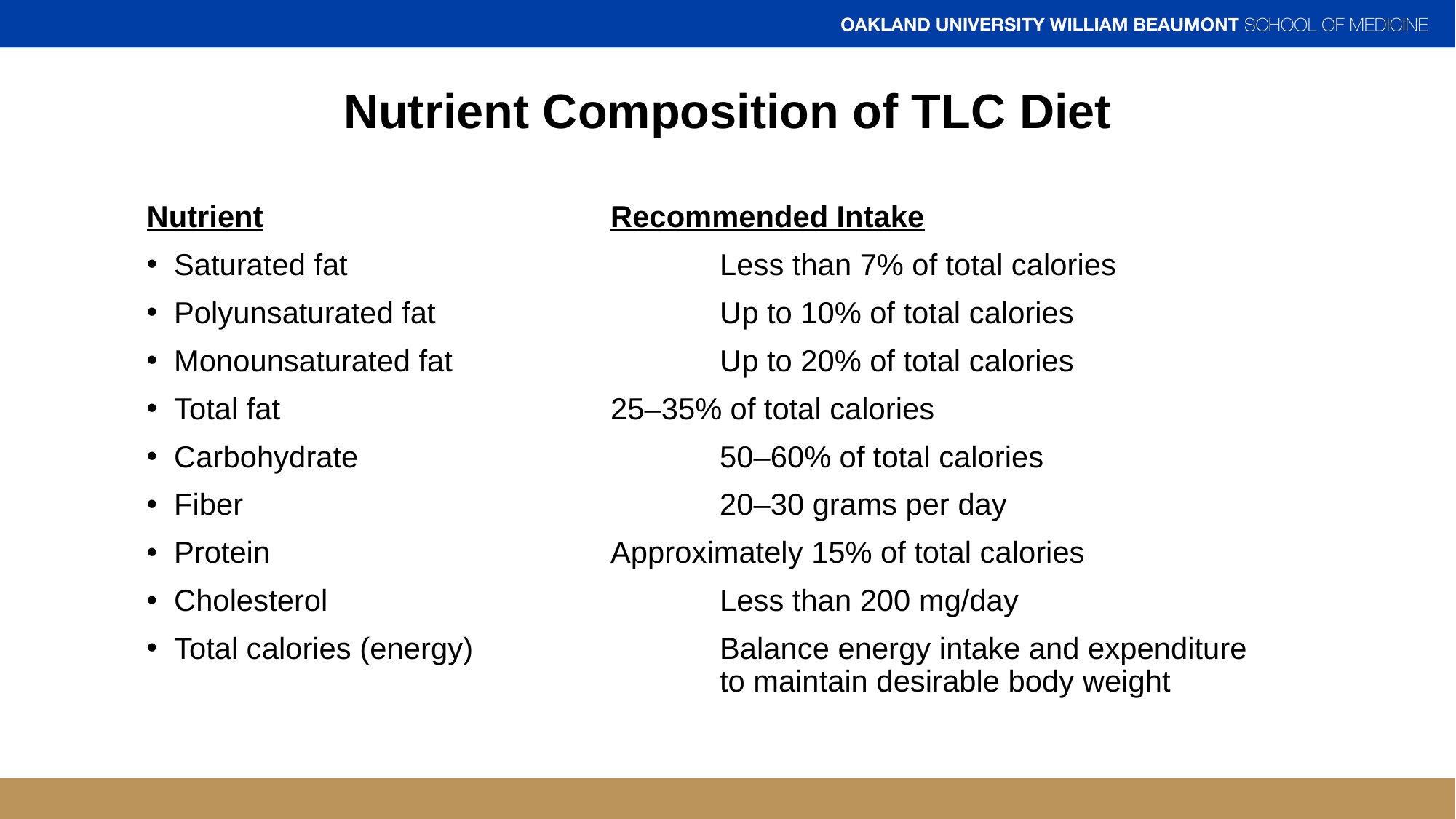

Nutrient Composition of TLC Diet
Nutrient				Recommended Intake
Saturated fat				Less than 7% of total calories
Polyunsaturated fat			Up to 10% of total calories
Monounsaturated fat 			Up to 20% of total calories
Total fat				25–35% of total calories
Carbohydrate				50–60% of total calories
Fiber					20–30 grams per day
Protein				Approximately 15% of total calories
Cholesterol				Less than 200 mg/day
Total calories (energy)			Balance energy intake and expenditure 							to maintain desirable body weight

## Slide 9
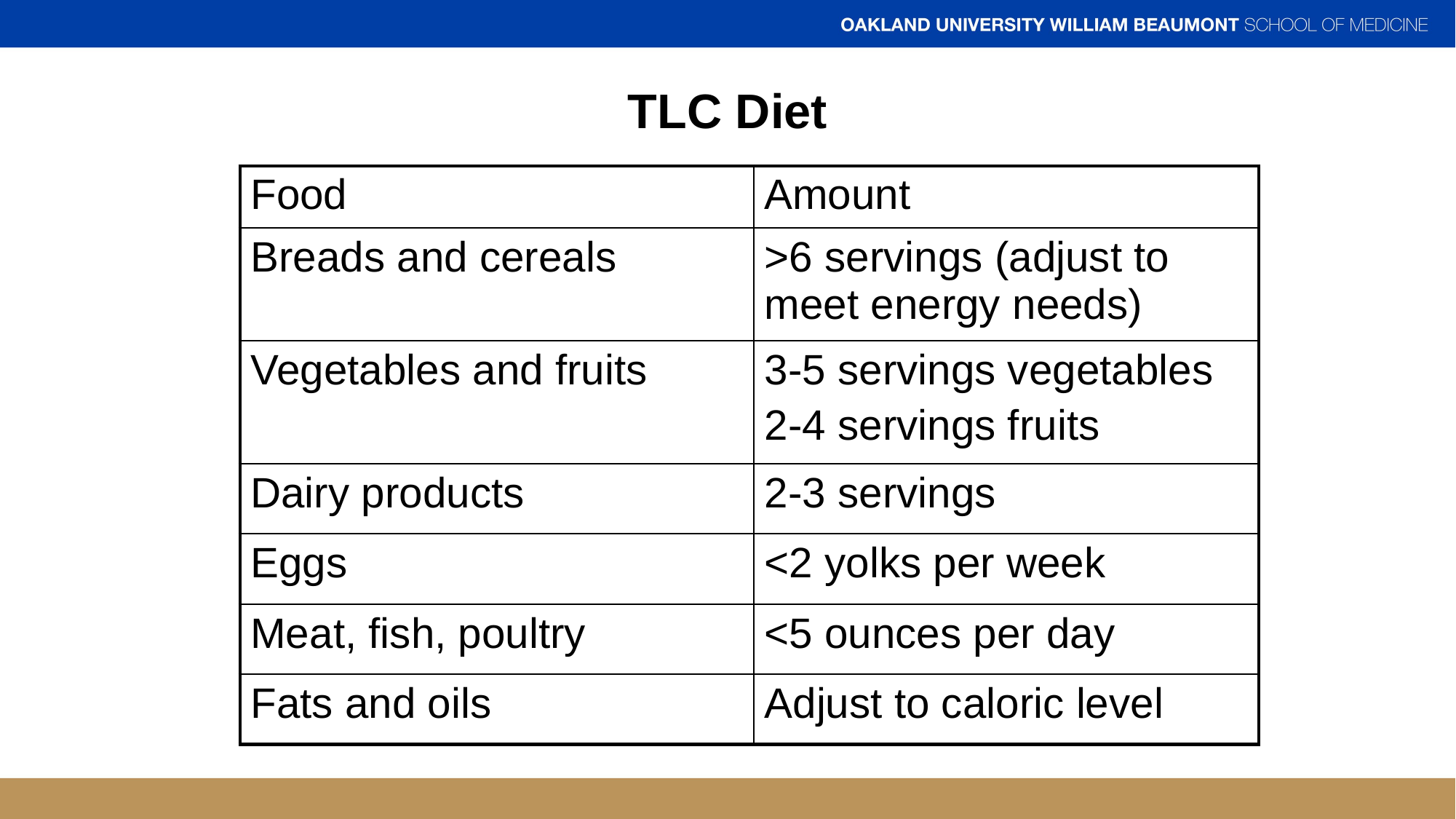

TLC Diet
| Food | Amount |
| --- | --- |
| Breads and cereals | >6 servings (adjust to meet energy needs) |
| Vegetables and fruits | 3-5 servings vegetables 2-4 servings fruits |
| Dairy products | 2-3 servings |
| Eggs | <2 yolks per week |
| Meat, fish, poultry | <5 ounces per day |
| Fats and oils | Adjust to caloric level |

## Slide 10
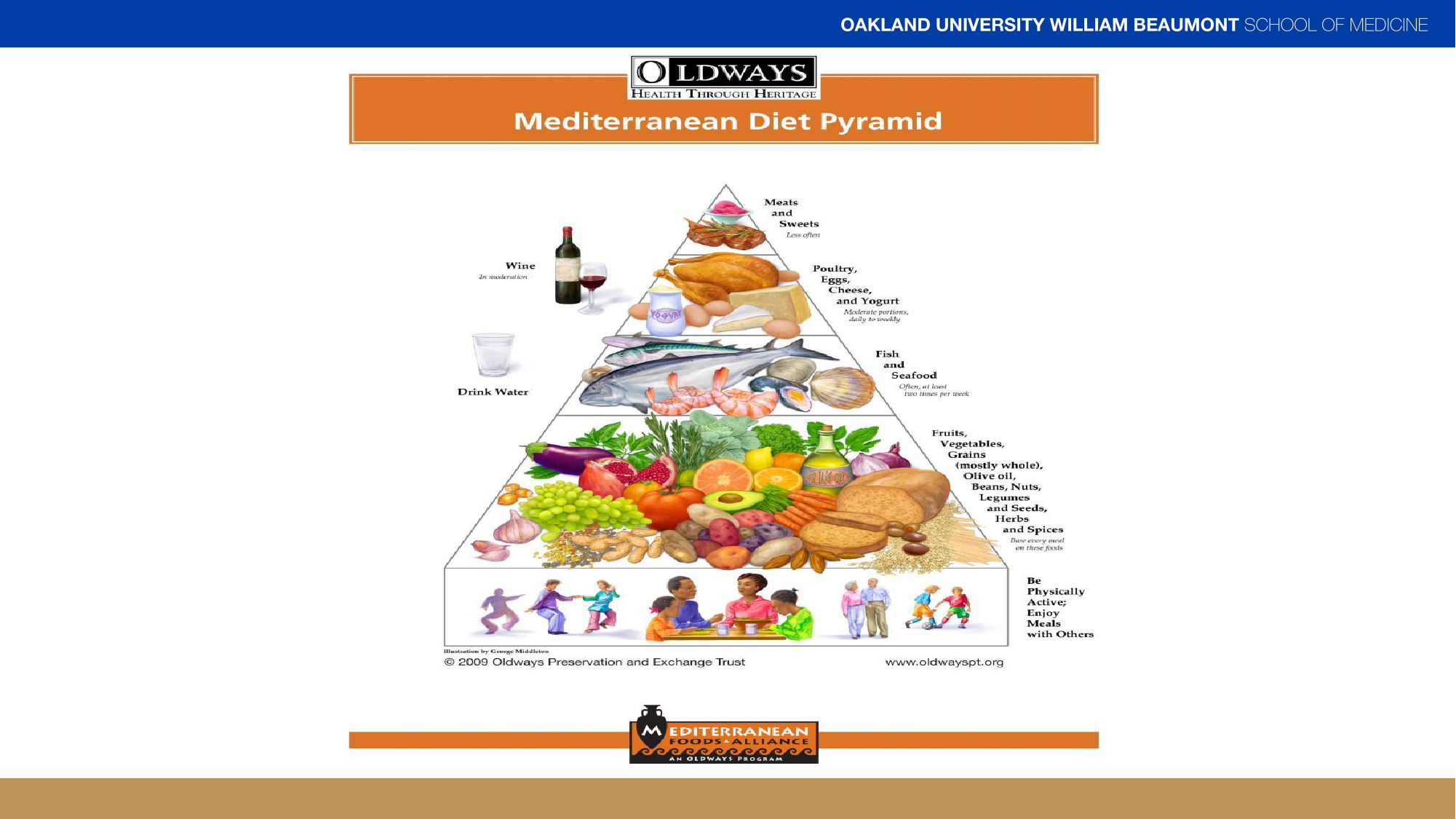

## Slide 11
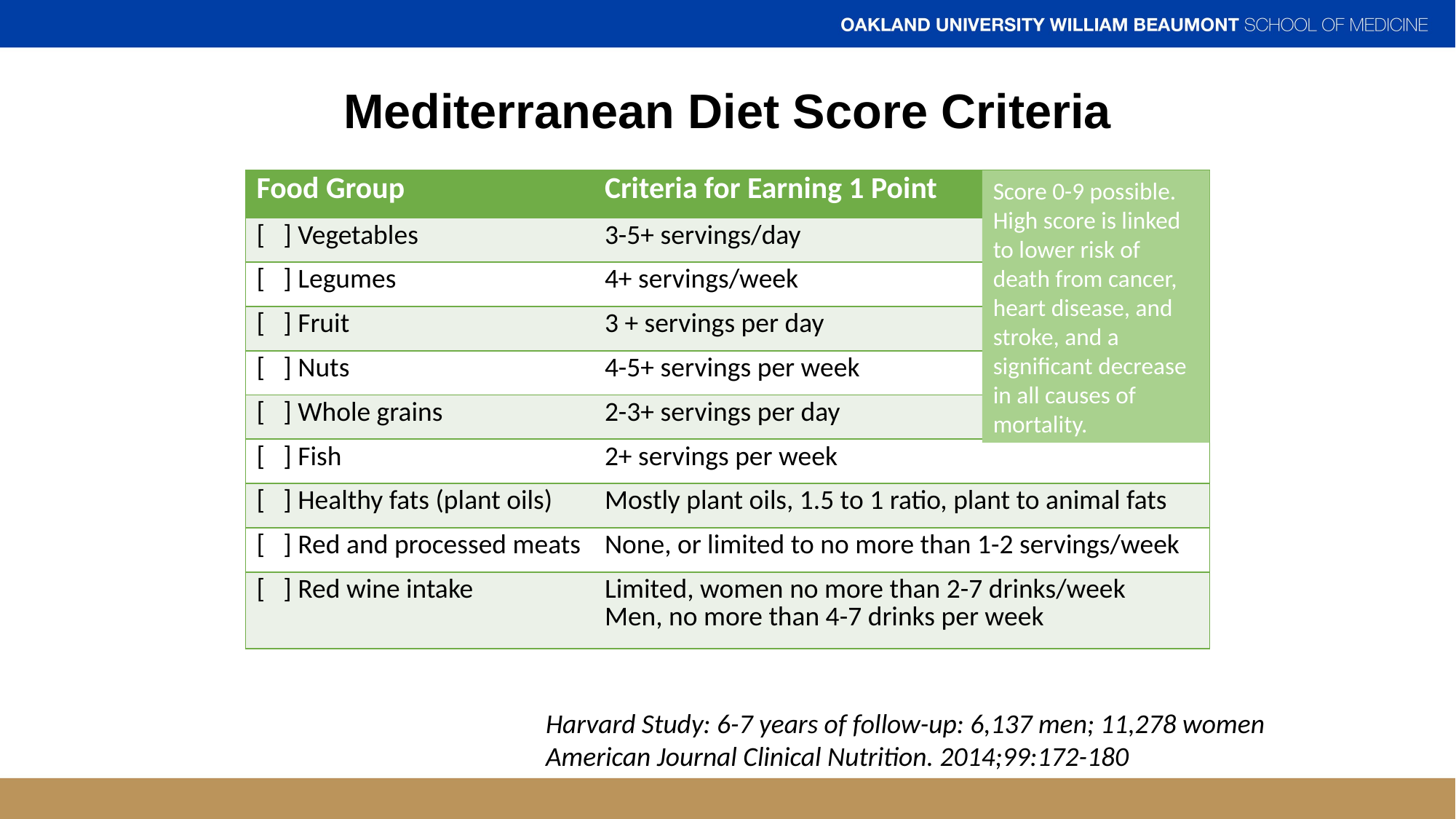

Mediterranean Diet Score Criteria
| Food Group | Criteria for Earning 1 Point |
| --- | --- |
| [ ] Vegetables | 3-5+ servings/day |
| [ ] Legumes | 4+ servings/week |
| [ ] Fruit | 3 + servings per day |
| [ ] Nuts | 4-5+ servings per week |
| [ ] Whole grains | 2-3+ servings per day |
| [ ] Fish | 2+ servings per week |
| [ ] Healthy fats (plant oils) | Mostly plant oils, 1.5 to 1 ratio, plant to animal fats |
| [ ] Red and processed meats | None, or limited to no more than 1-2 servings/week |
| [ ] Red wine intake | Limited, women no more than 2-7 drinks/week Men, no more than 4-7 drinks per week |
Score 0-9 possible. High score is linked to lower risk of death from cancer, heart disease, and stroke, and a significant decrease in all causes of mortality.
Harvard Study: 6-7 years of follow-up: 6,137 men; 11,278 women
American Journal Clinical Nutrition. 2014;99:172-180

## Slide 12
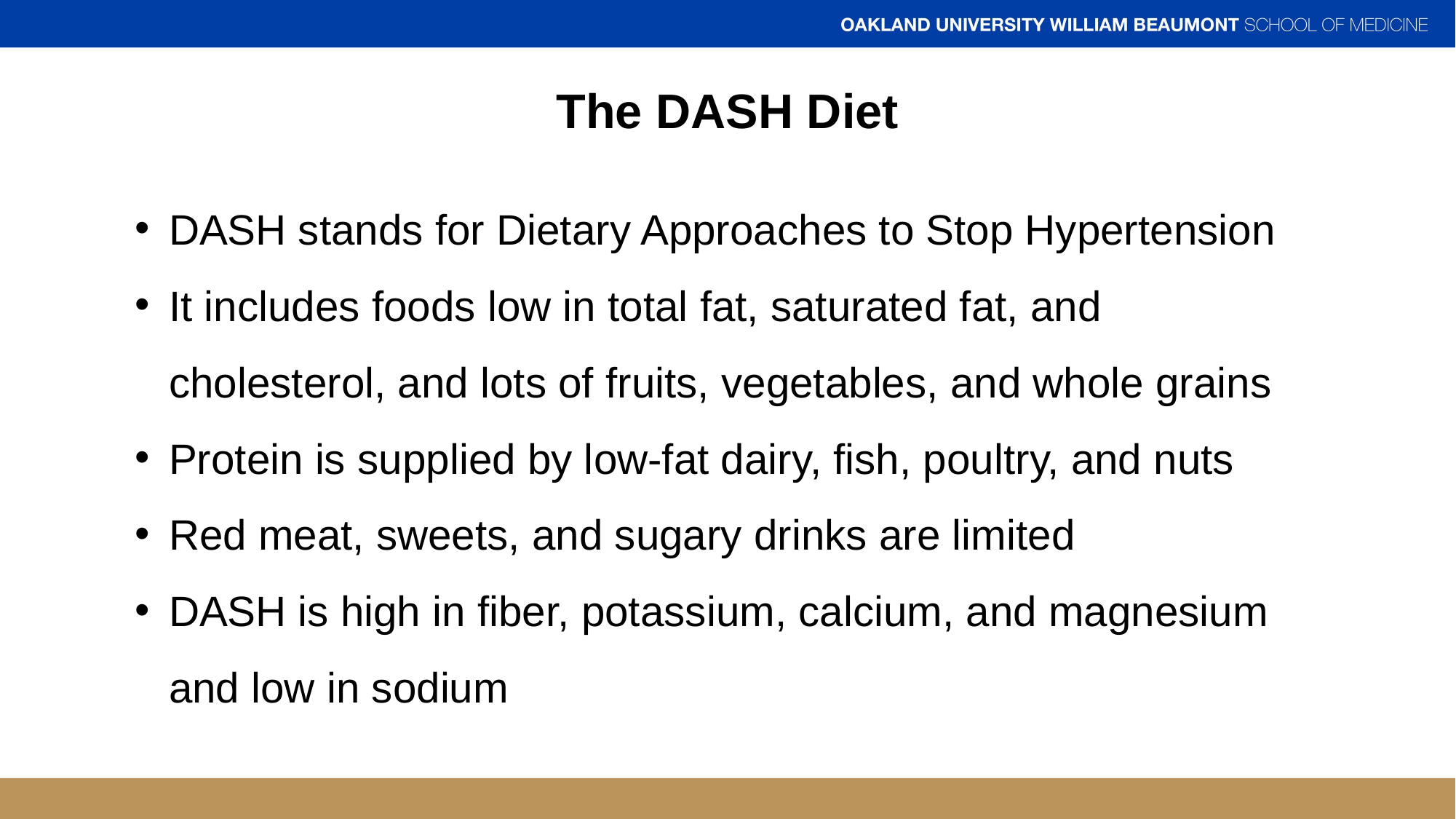

The DASH Diet
DASH stands for Dietary Approaches to Stop Hypertension
It includes foods low in total fat, saturated fat, and cholesterol, and lots of fruits, vegetables, and whole grains
Protein is supplied by low-fat dairy, fish, poultry, and nuts
Red meat, sweets, and sugary drinks are limited
DASH is high in fiber, potassium, calcium, and magnesium and low in sodium

## Slide 13
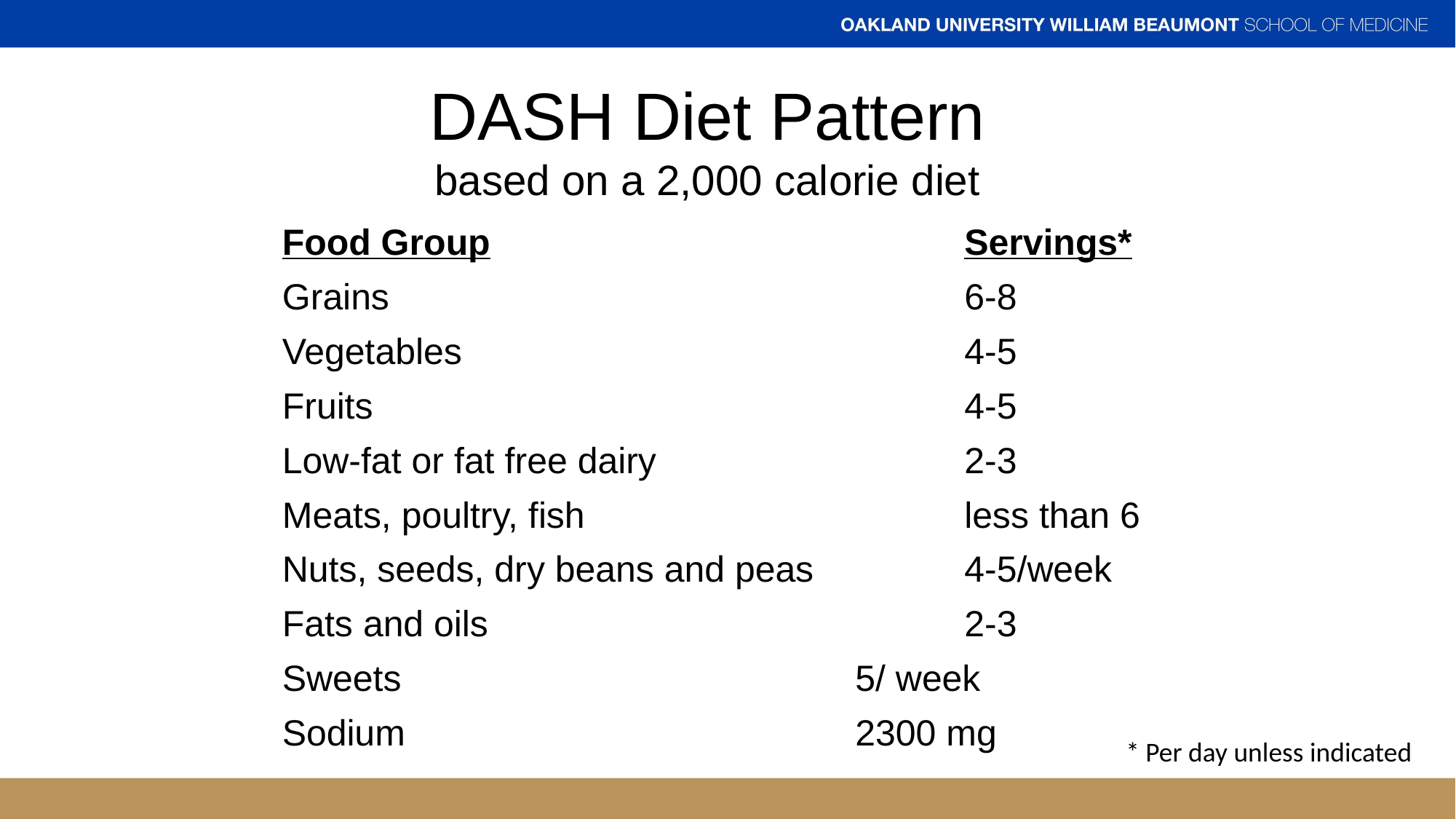

DASH Diet Pattern
based on a 2,000 calorie diet
Food Group					Servings*
Grains						6-8
Vegetables					4-5
Fruits						4-5
Low-fat or fat free dairy			2-3
Meats, poultry, fish				less than 6
Nuts, seeds, dry beans and peas		4-5/week
Fats and oils					2-3
Sweets					5/ week
Sodium					2300 mg
* Per day unless indicated

## Slide 14
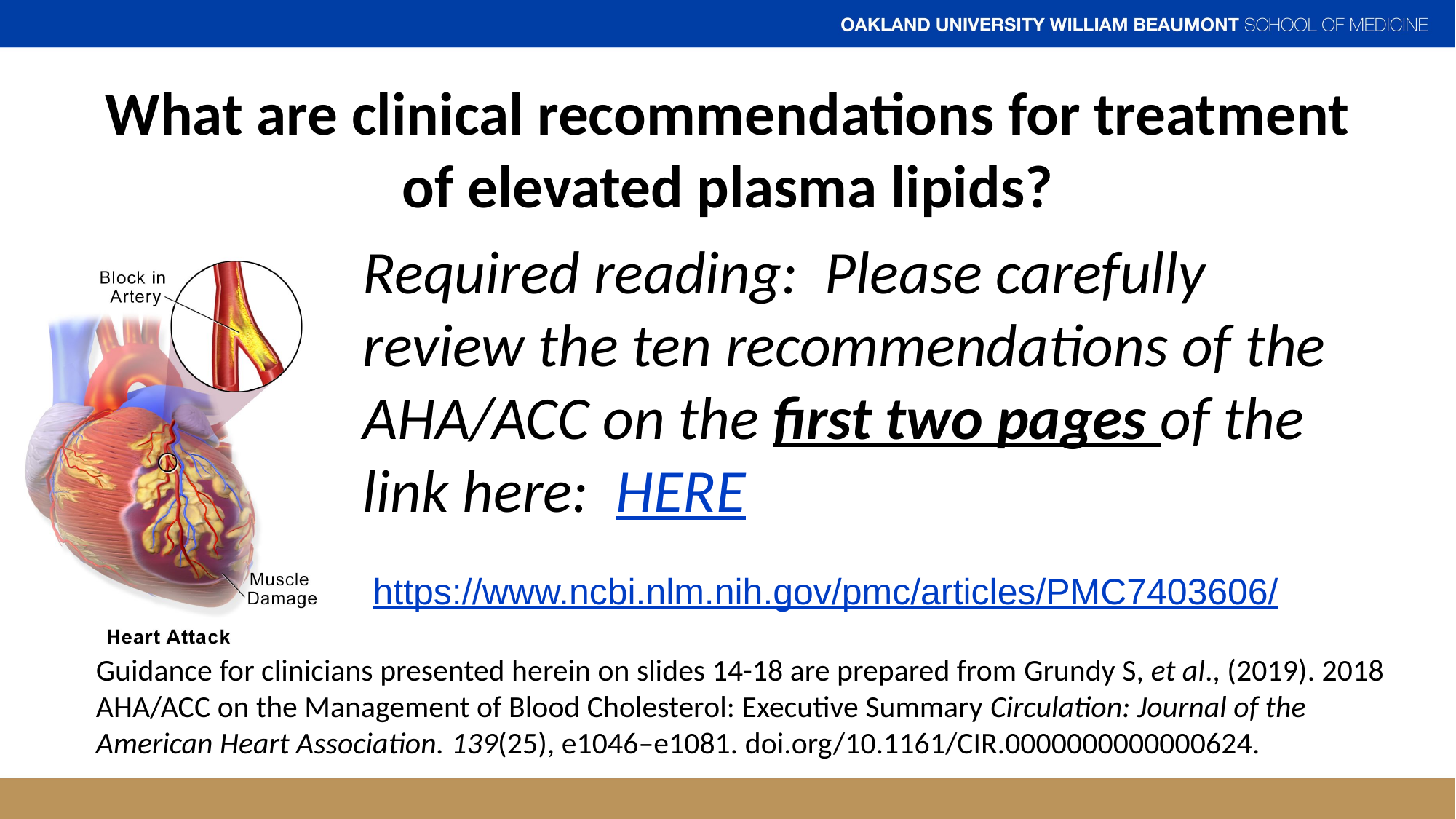

What are clinical recommendations for treatment of elevated plasma lipids?
Required reading: Please carefully review the ten recommendations of the AHA/ACC on the first two pages of the link here: HERE
 https://www.ncbi.nlm.nih.gov/pmc/articles/PMC7403606/
Guidance for clinicians presented herein on slides 14-18 are prepared from Grundy S, et al., (2019). 2018 AHA/ACC on the Management of Blood Cholesterol: Executive Summary Circulation: Journal of the American Heart Association. 139(25), e1046–e1081. doi.org/10.1161/CIR.0000000000000624.

## Slide 15
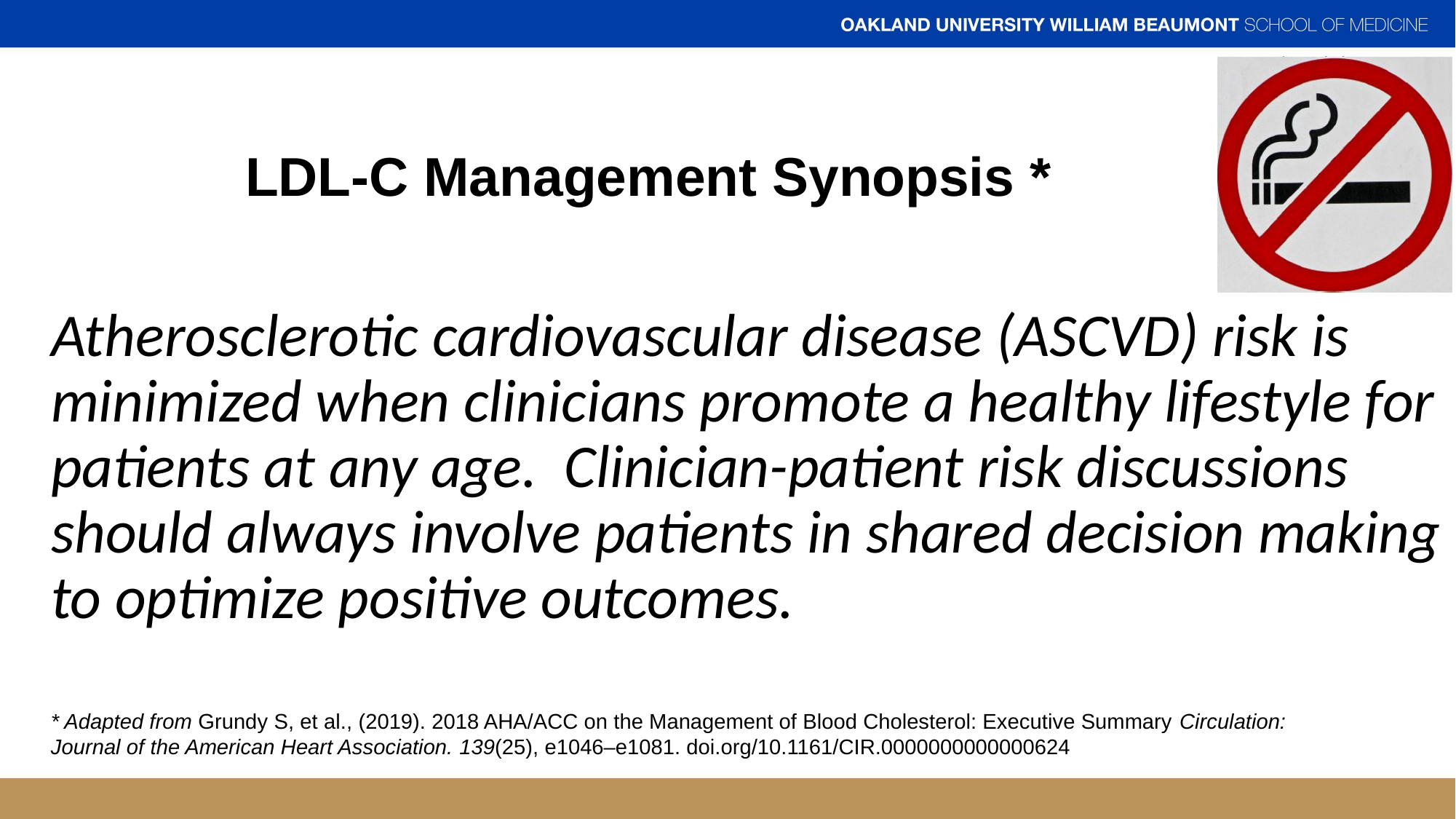

LDL-C Management Synopsis *
Atherosclerotic cardiovascular disease (ASCVD) risk is minimized when clinicians promote a healthy lifestyle for patients at any age. Clinician-patient risk discussions should always involve patients in shared decision making to optimize positive outcomes.
* Adapted from Grundy S, et al., (2019). 2018 AHA/ACC on the Management of Blood Cholesterol: Executive Summary Circulation: Journal of the American Heart Association. 139(25), e1046–e1081. doi.org/10.1161/CIR.0000000000000624

## Slide 16
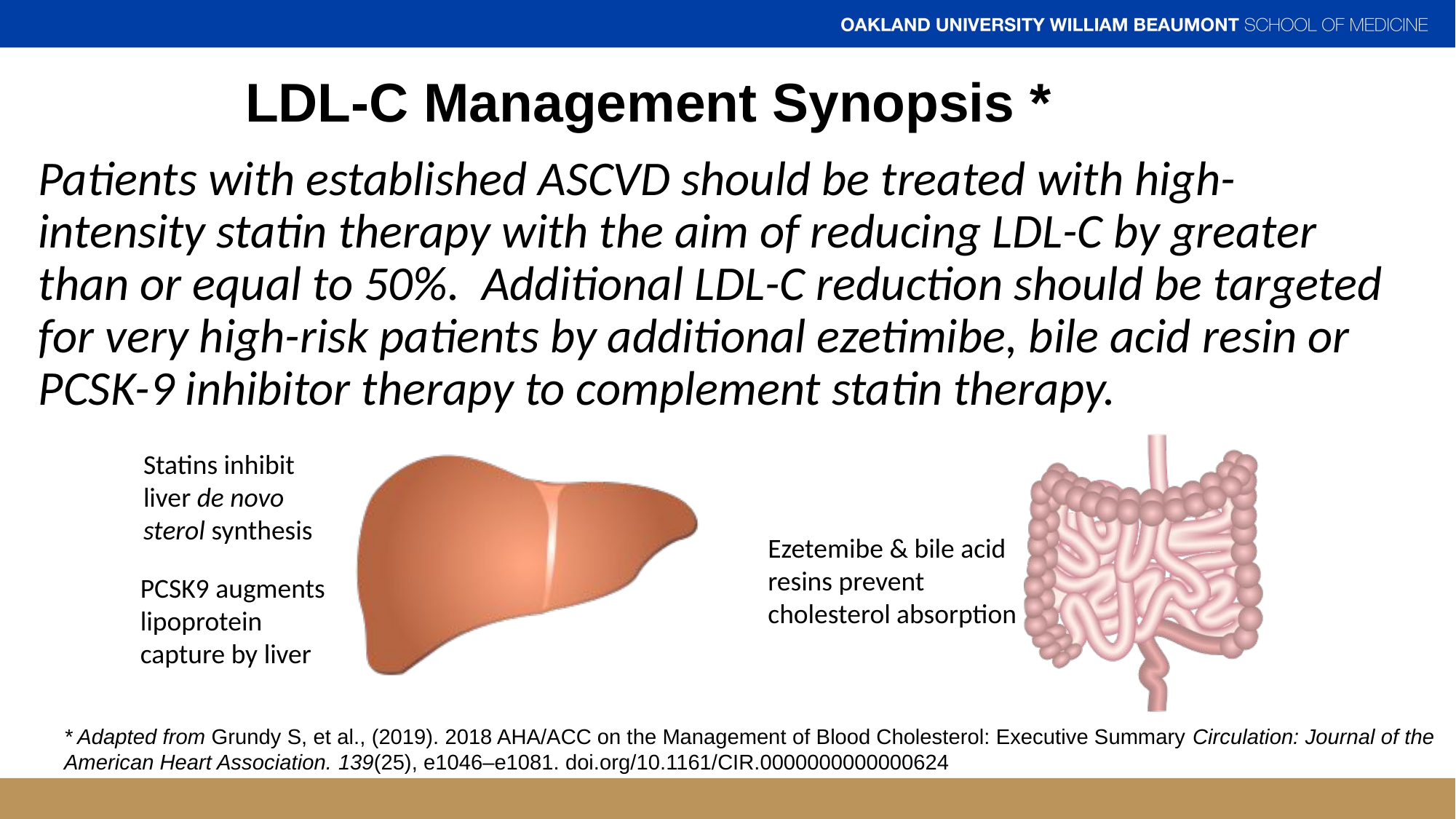

LDL-C Management Synopsis *
Patients with established ASCVD should be treated with high-intensity statin therapy with the aim of reducing LDL-C by greater than or equal to 50%. Additional LDL-C reduction should be targeted for very high-risk patients by additional ezetimibe, bile acid resin or PCSK-9 inhibitor therapy to complement statin therapy.
Statins inhibit liver de novo sterol synthesis
Ezetemibe & bile acid resins prevent cholesterol absorption
PCSK9 augments lipoprotein capture by liver
* Adapted from Grundy S, et al., (2019). 2018 AHA/ACC on the Management of Blood Cholesterol: Executive Summary Circulation: Journal of the American Heart Association. 139(25), e1046–e1081. doi.org/10.1161/CIR.0000000000000624

## Slide 17
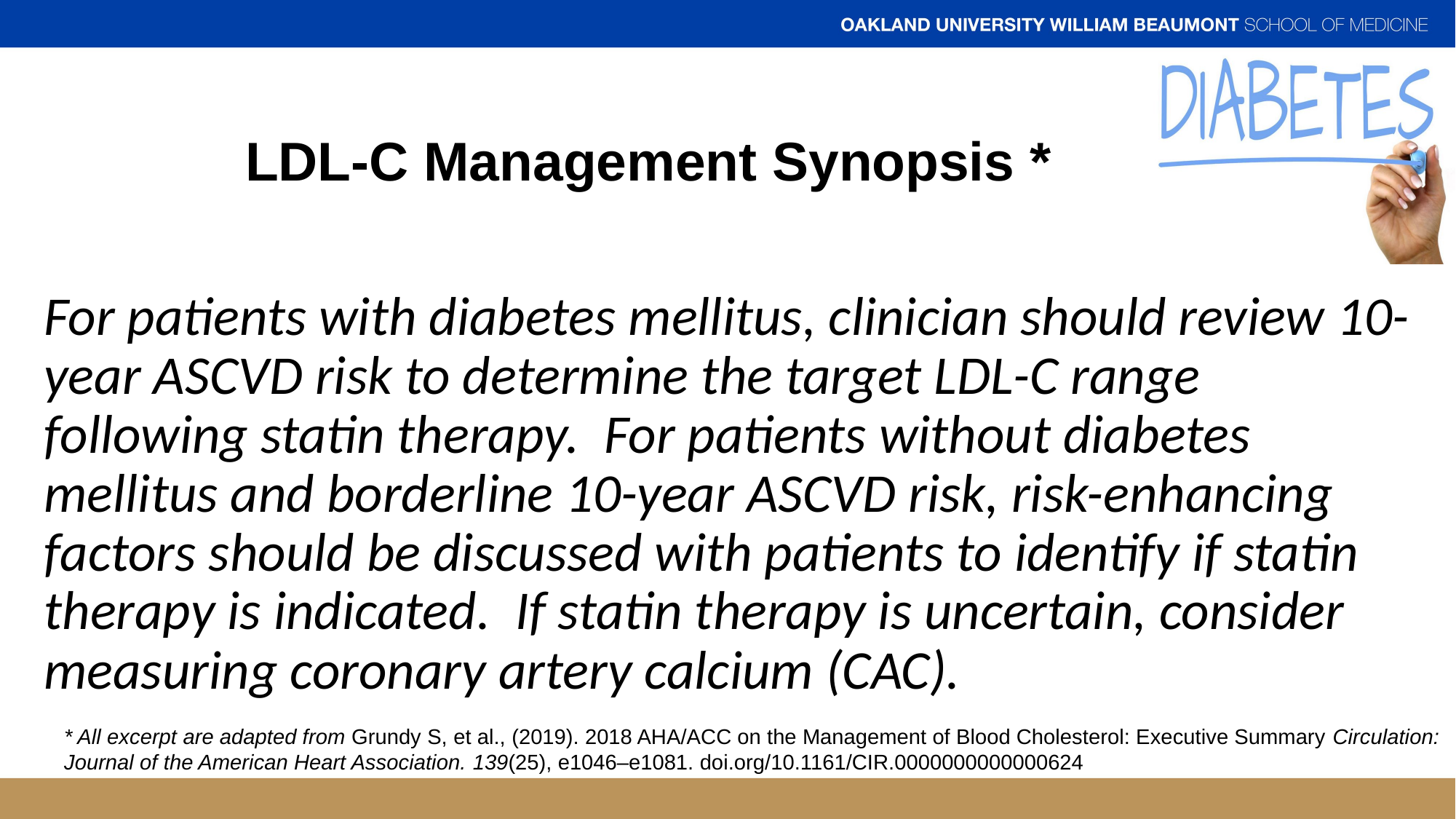

LDL-C Management Synopsis *
For patients with diabetes mellitus, clinician should review 10-year ASCVD risk to determine the target LDL-C range following statin therapy. For patients without diabetes mellitus and borderline 10-year ASCVD risk, risk-enhancing factors should be discussed with patients to identify if statin therapy is indicated. If statin therapy is uncertain, consider measuring coronary artery calcium (CAC).
* All excerpt are adapted from Grundy S, et al., (2019). 2018 AHA/ACC on the Management of Blood Cholesterol: Executive Summary Circulation: Journal of the American Heart Association. 139(25), e1046–e1081. doi.org/10.1161/CIR.0000000000000624

## Slide 18
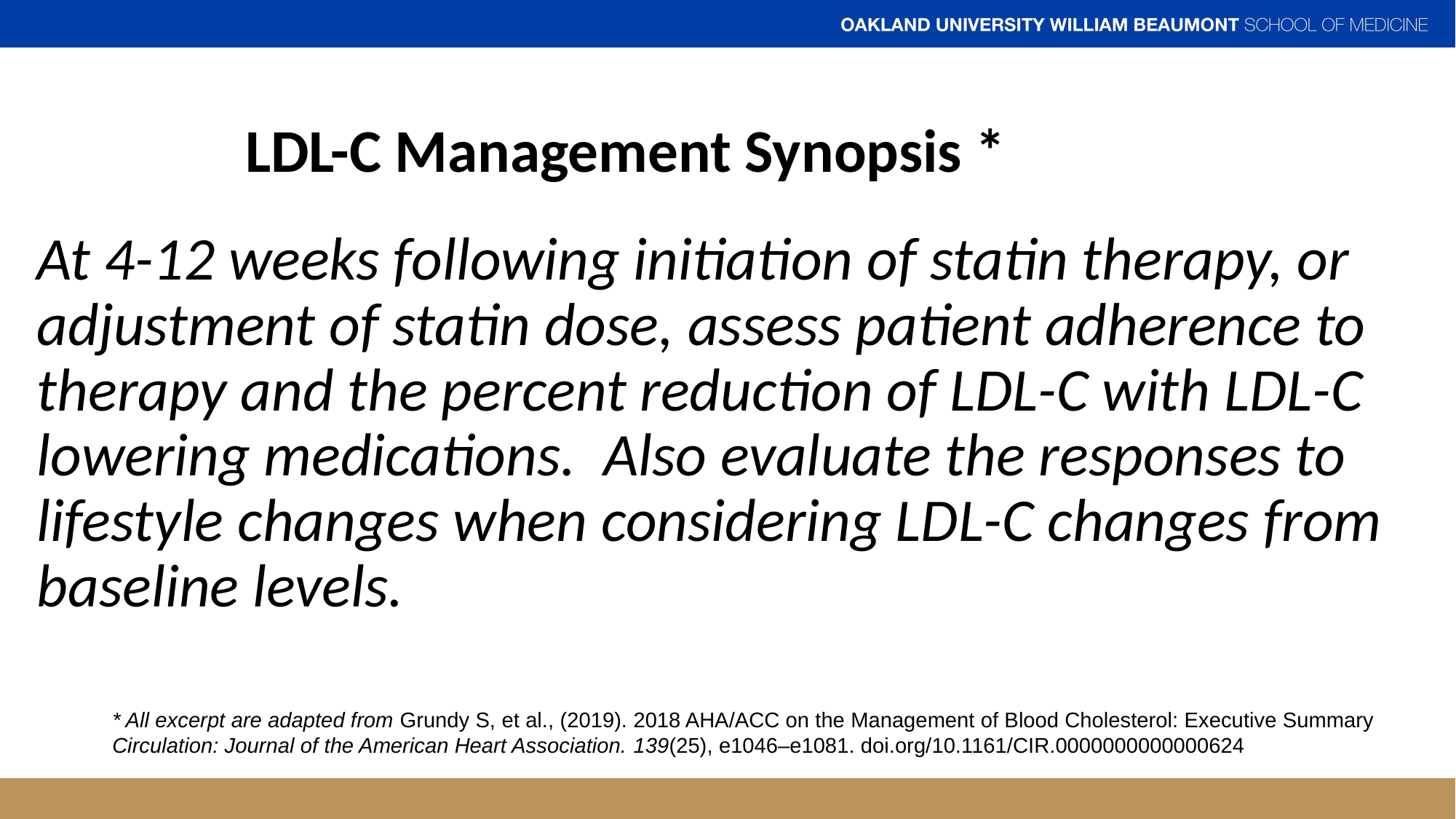

LDL-C Management Synopsis *
At 4-12 weeks following initiation of statin therapy, or adjustment of statin dose, assess patient adherence to therapy and the percent reduction of LDL-C with LDL-C lowering medications. Also evaluate the responses to lifestyle changes when considering LDL-C changes from baseline levels.
* All excerpt are adapted from Grundy S, et al., (2019). 2018 AHA/ACC on the Management of Blood Cholesterol: Executive Summary Circulation: Journal of the American Heart Association. 139(25), e1046–e1081. doi.org/10.1161/CIR.0000000000000624
