# Supplementary material for: Foundational and Clinical Science Integration in a Team-Based Learning Module Modeling Care of a Patient With Dyslipidemia
Source: MedEdPORTAL. 2024 Apr 9;20:11397. doi: 10.15766/mep_2374-8265.11397 (PMC11001791; doi:10.15766/mep_2374-8265.11397)
Supplement: Supplementary file 1 — Preparation Resources.pptxReadiness Assurance Test.docxRAT Question Appeal Form.docxApplication Exercises.docxFacilitator Guide.docx [file mep_2374-8265.11397-s001.zip › B. Readiness Assurance Test.docx]

ATTENTION, STUDENTS: If you are accessing this material BEFORE it is used in your course, please do NOT read this document prior to the class session. An answer key is included in this module, which is designed to lead you through a learning experience that reinforces your knowledge of the content. Early review or dissemination of this material to others will diminish the learning opportunity and be considered academic misconduct.

**APPENDIX B. READINESS ASSURANCE TEST**

**iRAT:** 9 minutes

**tRAT:** 12 minutes plus 10 minutes for discussion (note that the use of more challenging RAT questions (see below) may require more time for tRAT discussion)

1. **Which of the following interventions for metabolic syndrome is best to consider for the treatment of patients in all age groups?**
2. Aspirin monotherapy
3. Combination statin and ezetimibe therapy
4. Lifestyle therapy
5. Statin monotherapy
6. **A 60-year-old patient with atherosclerotic cardiovascular disease (ASCVD) is prescribed a statin. The aim of statin therapy is to use up to the maximum tolerated dose of statins to lower LDL-C to the target range. What is the desired LDC-C target range following statin therapy for patients with ASCVD?**
7. ≥90% of initial LDL-C
8. ≥50% of initial LDL -C
9. ≥20% of initial LDL -C
10. ≥5% of initial LDL -C
11. **During a physical examination by her pediatrician, a 9-year-old girl is found to have a xanthoma on her Achilles tendon. A lipid panel is ordered for the girl, which reveals an LDL-C value of 505 mg/dL. The pediatrician’s preliminary diagnosis is familial hypercholesterolemia.**

**What are the most likely genotypes of the genes responsible for controlling LDL-C levels in the girl’s parents?**

A. Both parents are heterozygous for a pathogenic mutation in the same LDL-C regulatory gene.

B. Neither parent carries a pathogenic mutation in these genes because the girl’s condition likely arose as the result of a *de novo* mutation.

C. Only one parent carries a pathogenic mutation in a gene controlling LDL-C levels.

D. The girl’s condition is polygenic in origin, resulting from the inheritance of multiple mutations in a number of genes controlling LDL-C levels.

1. **A 48-year-old female has recently been diagnosed with hypercholesterolemia. She indicates that she would like to try medical nutrition therapy for 6 weeks to lower her blood cholesterol levels before she will try any lipid lowering drug therapy.**

**If you were to recommend trying the DASH dietary pattern, based on the food intake recommendations, which of the following would be a specific target to increase in her diet?**

1. Iron
2. Magnesium
3. Vitamin D
4. Thiamin
5. **A 48-year-old female has recently been diagnosed with hypercholesterolemia. She indicates that she would like to try medical nutrition therapy for 6 weeks to lower her blood cholesterol levels before she will try any lipid lowering drug therapy.**

**If you were to recommend the Therapeutic Lifestyle Change diet approach, what type and percent of total calories (Kcals) of dietary lipid intake will you advise her to consume to help lower her LDL (low density lipoprotein) blood cholesterol level?**

1. Reduce saturated fat intake to 20% of her total caloric intake
2. Reduce all fat intake to 10% of her total caloric intake
3. Reduce saturated fat intake to <7% of her total caloric intake
4. Reduce polyunsaturated fat intake to <5% of her total caloric intake
5. **At what point should adherence to changes in lifestyle and effects of LDL-C lowering medications be assessed by measurement of fasting lipids and appropriate safety indicators (liver function, muscle enzymes)?**
6. After the patient has demonstrated adequate changes to their lifestyle or shown medication compliance for at least 3 months
7. Every 3 months until the LDL-C goal is achieved based on shared decision-making goals between patient and physician
8. 4-12 weeks after statin initiation or dose adjustment and every 3-12 months thereafter based on need to assess adherence or safety
9. Next routine office visit or at the time of the patient’s next comprehensive physical

**Answer Key:**

1. **C**
2. **B**
3. **A**
4. **B**
5. **C**
6. **C**

**RAT Question Rationales**

1. Option C (Lifestyle therapy) is the best answer.  A healthy lifestyle in younger individuals can reduce future risk, and for 20-39-year-old patients, a consideration of lifestyle factors is helpful in fostering future dialogue about risk.  Aspirin, statin or statin and ezetimibe therapy will be appropriate for a more limited group of patients, depending on known side-effects and risk-benefit of specific pharmacotherapy.
2. Option B (50% of initial patient levels) is correct.  According to guidance provided in the 2018 AHA/ACC on the Management of Blood Cholesterol, the target for LDL-C reduction should be ≥50% of the patient’s original (high) LDL-C.  A target reduction of ≥90% of initial levels would not be sufficient to significantly lower CVD risk.  A target range of ≥20% of the patient’s original LDL-C is not realistic using statin therapy alone. Use of a combination therapy or a PCSK-9 inhibitor would be suggested to achieve higher efficacy of LDL-C reduction.
3. Option A (heterozygosity of both parents) is correct. The presence of xanthoma in a nine-year-old child having LDL-C levels above 500 mg/dL strongly suggests that the child is homozygous for a pathogenic mutation in an LDL-C regulatory gene (or is compound heterozygote, carrying two different pathogenic alleles of the same gene). The most likely explanation for the child’s homozygosity is that she inherits a mutant allele of the same LDL-C regulatory gene from each of her parents, who are both heterozygotes. *De novo* mutation of LDL-C regulatory genes during gametogenesis in both parents is formally possible, but would be significantly less likely than parental heterozygosity. The severity of the child’s condition is inconsistent with her being merely heterozygous for a pathogenic LDL-C regulatory gene mutation. Lastly, while polygenic hypercholesterolemia is associated with premature coronary heart disease, it would not be expected to cause xanthoma in a nine-year-old child.
4. Option B (Magnesium) is correct. The DASH diet eating pattern includes recommendations for the inclusion of food sources that are "high in potassium, calcium, and magnesium" [National Heart, Lung, and Blood Institute website. DASH eating plan. [www.nhlbi.nih.gov/education/dash-eating-plan](https://www.google.com/url?q=http://www.nhlbi.nih.gov/education/dash-eating-plan&sa=D&source=docs&ust=1690811156230917&usg=AOvVaw0mwFqY4o8VAkAS4HuNQ91S). Updated December 29, 2021. Accessed July 27, 2023].
5. Option C (Reduce saturated fat intake to <7% of her total caloric intake) is correct. The Therapeutic Lifestyle Change dietary pattern recommendations include "Less than 7 percent of your daily calories from saturated fat". [National Institutes of Health, National Heart, Lung, and Blood Institute. Your guide to lowering your cholesterol with TLC: Therapeutic Lifestyle Changes. [https://www.nhlbi.nih.gov/resources/your-guide-lowering-cholesterol-therapeutic-lifestyle-changes-tlc](https://www.google.com/url?q=https://www.nhlbi.nih.gov/resources/your-guide-lowering-cholesterol-therapeutic-lifestyle-changes-tlc&sa=D&source=docs&ust=1690811156236438&usg=AOvVaw3W2ZpXbPtEq54y_trM9ouw). Published December, 2005. Accessed July 27, 2023.]
6. Option C (4-12 weeks after statin initiation or dose adjustment and every 3-12 months thereafter based on need to assess adherence or safety) is correct. Answer A is ambiguous and does not give guidance as to what is most appropriate. Answer B is consistent with the 2018 guidelines for management of blood cholesterol. Answer C is too soon to assess lifestyle changes and too long after medication initiation. Answer D is too frequent.

**Additional RAT Questions**

NOTE: Performance data reported in the Education Summary Report correspond to Questions 1-6 above. We provide a number of additional, more challenging RAT questions with rationales below that may be used at the discretion of facilitators.

1. **The treatment of a 48-year-old man with an LDL-C level of 200 mg/dL and a history of obesity and diabetes would benefit most by which of the following next steps?**
2. Calculation of the patient’s 10-year ASCVD risk to determine if statin therapy is indicated
3. Enrollment in a trial of non-statin therapy with repeat lipid levels in three months
4. Recommendation to start a “keto” diet and recheck lipids in six months
5. Recommendation to start high intensity statin therapy

Rationale: Option D (Recommendation to start high intensity statin therapy) is correct. The keto diet is associated with higher LDL levels based on recent studies. In patients with an LDL-C >190 mg/dL and a history of diabetes, starting statin therapy with a high intensity statin is recommended regardless of ASCVD risk. Non statin therapy would not be considered first line treatment in a patient with diabetes.

1. **A 60-year-old woman with no history of diabetes mellitus and a current LDL-C of 110 mg/dL is concerned about possible statin therapy side-effects that might interfere with her active lifestyle. The patient’s 10-year ASCVD risk is 15.0%. What additional assessment best provides decision-making support, given the patient’s concerns?**

1. Coronary artery calcium (CAC) score
2. HbA1C determination
3. Individualized estimate of daily dietary cholesterol
4. Review of medical history of second-degree family members

Rationale: Option A [Coronary artery calcium (CAC) score] is correct. According to the 2018 ASCVD guidance, the CAC score can be helpful in situations that suggest value to solidify advice to patients. HbA1C is a measure of blood glucose and would not be directly of value. Dietary cholesterol estimation can provide some value, but will not be as informative as LDL-C values that provide more direct information. A review of the medical histories of second-degree relatives will not be as informative as that of first-degree relatives, for example.

**9. A 30-year-old woman meets with her primary care physician for an annual evaluation. The physician considers that the most effective means to facilitate a meaningful clinician-patient risk discussion is to review which of the following?**

A. Consideration of drug treatment options

B. Discussion of the benefit of regular exercise

C. Assessment of patient’s lifetime risk

D. Review of foods to avoid

Rationale: Option C (Assessment of patient’s lifetime risk) is correct. In patients in this age group, providing an individualized assessment of lifetime risk, emphasizing lifestyle is most effective. No patient information provided suggests the need for exercise plans, drug treatment, or dietary concerns.

1. **A 65-year-old man meets with his primary care physician for a new patient visit. The patient history reveals a long-term concern about a family history of ASCVD. The patient has been diagnosed with Type II diabetes mellitus, has obesity, and is rated as very high risk for ASCVD based upon a history of multiple prior ASCVD events. The patient has an LDL-C measured as 100 mg/dL.**

**What is the best intervention for the physician to consider if the patient’s current statin is already at a maximally tolerated dose?**

1. Consider ezetimibe to augment current medication
2. Develop a lifestyle-based plan with the aim of reducing LDL-C by 10%
3. Discuss the benefits of caloric control
4. Discuss the benefits of exercise therapy
5. Reduce statin therapy to moderate intensity dosing

Rationale: Option A (Consider ezetimibe to augment current medication) is correct. Ezetimibe acts by an entirely different mechanism to reduce LDL-C. The target range for this patient’s LDL-C is ≤ 70 mg/dL given the very high risk for ASCVD. Lifestyle and dietary interventions are helpful but not likely to produce a 30% reduction in current LDL-C levels by themselves. Reducing statin therapy from a maximally tolerated dose will be expected to increase and not decrease LDL-C.

1. **An 18-year-old man diagnosed with familial hypercholesterolemia is treated with the maximal tolerated dose of a statin. However, the treatment fails to achieve recommended LDL-C levels. His physician recommends treatment with a PCSK9 inhibitor. Which of the following best describes its mechanism of action?**
2. Inhibition of hormone-sensitive lipase activity
3. Reduction of LDL receptor degradation
4. Reduction of LDL receptor internalization by endocytosis
5. Repression of ApoB gene expression

Rationale: Option B (Reduction of LDL receptor degradation) is correct. The PCSK9 gene product interacts with the LDL receptor and promotes receptor degradation by the lysosome following endocytosis. Inhibitors of the PCSK9 gene product prevent its association with the LDL receptor, resulting in reductions in LDL receptor degradation. As a result, serum LDL-C levels are expected to decrease as more LDL receptors are recycled to the cell surface, thereby increasing LDL-C binding and internalization.

1. **A 48-year-old female has recently been diagnosed with hypercholesterolemia. She indicates that she would like to try medical nutrition therapy for 6 weeks to lower her blood cholesterol levels before she will try any lipid lowering drug therapy.**

**If her physician were to recommend the Therapeutic Lifestyle Change dietary pattern approach, by lowering her dietary intake of saturated fat to 7% of her total calories, what percent reduction of LDL-C would be expected for this patient in 6 weeks?**

1. 2%
2. 5%
3. 8%
4. 12%
5. 15%

Rationale: Option C (8%) is correct. Based on the published literature, the expected reduction in LDL-C would be 8%-10%.
