# Supplementary material for: Foundational and Clinical Science Integration in a Team-Based Learning Module Modeling Care of a Patient With Dyslipidemia
Source: MedEdPORTAL. 2024 Apr 9;20:11397. doi: 10.15766/mep_2374-8265.11397 (PMC11001791; doi:10.15766/mep_2374-8265.11397)
Supplement: Supplementary file 1 — Preparation Resources.pptxReadiness Assurance Test.docxRAT Question Appeal Form.docxApplication Exercises.docxFacilitator Guide.docx [file mep_2374-8265.11397-s001.zip › C. RAT Question Appeal Form.docx]

**APPENDIX C. TBL TEAM READINESS ASSURANCE QUESTION APPEAL FORM**

*The appeal must be submitted to (insert relevant email address) within 24 hours after the TBL session. Appeals must be submitted AFTER the TBL activity. Forms submitted during the session will not be reviewed.*

Team Number:

Team Member Names:

1.

2.

3.

4.

5.

6.

7.

TBL Session Name:

tRAT question number:

Describe the issue you found with the question (e.g. wording or content). Please make sure to reference the material upon which your argument is based:

Faculty Facilitator Response to Appeal:
