# Supplementary material for: Foundational and Clinical Science Integration in a Team-Based Learning Module Modeling Care of a Patient With Dyslipidemia
Source: MedEdPORTAL. 2024 Apr 9;20:11397. doi: 10.15766/mep_2374-8265.11397 (PMC11001791; doi:10.15766/mep_2374-8265.11397)
Supplement: Supplementary file 1 — Preparation Resources.pptxReadiness Assurance Test.docxRAT Question Appeal Form.docxApplication Exercises.docxFacilitator Guide.docx [file mep_2374-8265.11397-s001.zip › D. Application Exercises.docx]

ATTENTION, STUDENTS: If you are accessing this material BEFORE it is used in your course, please do NOT read this document prior to the class session. An answer key is included in this module, which is designed to lead you through a learning experience that reinforces your knowledge of the content. Early review or dissemination of this material to others will diminish the learning opportunity and be considered academic misconduct.

**APPENDIX D. DYSLIPIDEMIA TBL APPLICATION EXERCISES**

**Application Exercise #1** (10 minutes with 10 minutes for discussion)

Gloria, a 57-year-old woman is a new patient reporting to a *primary care physician* for an initial visit. Her chief complaint is that she frequently experiences pain in her chest, typically after eating large meals. Her BMI is 31 and she reports smoking approximately one pack of “light” filtered cigarettes per week. Gloria reports that she leads a rather sedentary lifestyle as an office worker who gets little exercise.

Based on the initial findings, the physician reasons that Gloria is likely to be suffering from a dyslipidemia.

Laboratory values of a fasting lipid profile are as follows:

| Test | Result | Recommended Range |
| --- | --- | --- |
| Total Cholesterol | 300 mg/dL | Desirable, less than 200 mg/dL |
| High-density Lipoprotein (HDL) Cholesterol | 45 mg/dL | Desirable range (for age), 40-59 mg/dL |
| Low-density Lipoprotein (LDL) Cholesterol | TBD (227 mg/dL calculated) | Desirable, less than 130 mg/dL |
| Triglycerides | 140 mg/dL | Desirable, less than 150 mg/dL |

Physical exam did not indicate any evidence of vascular obstruction. No xanthomas or tendinous xanthomas were observed.

**Question:**

**For patients aged 40-75 years of age, risk discussions before statin therapy initiation should include review of major risk factors such as smoking, elevated blood pressure, LDL-C, and calculated risk of atherosclerotic cardiovascular disease.**

**What else should always be discussed with these patients?**

1. Costs of alternative therapies such as PCSK-9
2. Exercise routines to reduce muscle spasms
3. Family history of statin use
4. Importance of family support for compliance in statin therapy
5. *Patient preferences and values in shared-decision making*

**Application Exercise #2 (Nutrition)** (20 minutes with 10 minutes for discussion)

1. Review Gloria’s dietary intake information below.

2. Describe any dietary recommendations that could be discussed with this patient to help her reduce her blood cholesterol levels and cardiovascular disease risk.

3. Submit your team’s recommendations with your team number.

**Gloria’s Diet history** (based on usual dietary intake)

**Breakfast (home)**

2 eggs, fried with 1 teaspoon butter

2 slices of bacon

1 small avocado

1 cup green tea

**Lunch (work)**

1 Big Mac (no bun or sauce)

1 large French Fries

1 hard-boiled egg

12 oz Diet Cola

**Snack (work)**

1 handful of mixed nuts

5 oz cheese

1 cup yogurt (whole fat)

**Dinner (home)**

1 large portion of spaghetti (high protein) with meat sauce/tomato sauce

1 low carb dinner roll with garlic butter

1 small mixed green salad with Italian dressing (about 2 tablespoons)

1 8 oz glass Diet cola

1 small piece of Chocolate cake

**Snack (movie theater)**

1 small size buttered popcorn

12 oz Diet Cola

**Application Exercise #3 (Genetics)** (20 minutes with 10 minutes for discussion)

As described in the preparatory information, some dyslipidemias are caused by monogenic disorders, which is important information for treating not only the index patient, but potentially also the patient’s family members, while other dyslipidemias are polygenic.

Using the genetic family history that the physician has elicited from Gloria (below) and the attached chart of commonly-used pedigree symbols,

1. Work with your TBL team to draw a pedigree that most accurately reflects the family history obtained by the physician, and
2. State whether a recognizable pattern of inheritance exists within Gloria’s family, and if so, what type of pattern.
3. Which dyslipidemia is consistent with this pattern of inheritance, if one exists?
4. Photograph your team’s pedigree with answers to the above questions and your team number and submit your answer to the facilitators.

The physician interviews Gloria in order to elicit her genetic family history, details of which are provided below.

Gloria reports that her mother is 89-years-old, is in generally good health and is not overweight. Her father died many years ago at the age of 52 after suffering a heart attack. Gloria remembers that he was “real heavy and also took pills for cholesterol”. Gloria has a 55-year-old brother who is also overweight and has been taking pills for “bad cholesterol” for at least five years.

Upon further inquiry, the physician learns that Gloria’s dad had three brothers. One died in a car accident at the age of 35, one died from a heart attack at “about 50-years-old”, and her remaining uncle is 67-years-old. Gloria describes him as about 5’10” and 240 lbs. The brothers’ mom, Gloria’s grandmother, died many years ago at age 45 due to a “stroke”.

**Commonly Used Pedigree Symbols**

**
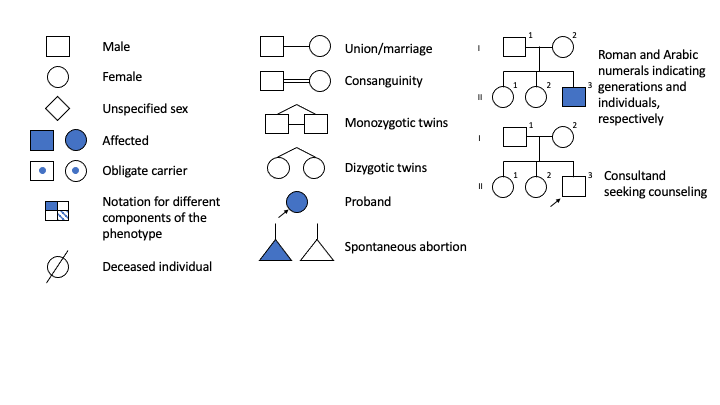
**

(author-owned image)
