# Supplementary material for: Foundational and Clinical Science Integration in a Team-Based Learning Module Modeling Care of a Patient With Dyslipidemia
Source: MedEdPORTAL. 2024 Apr 9;20:11397. doi: 10.15766/mep_2374-8265.11397 (PMC11001791; doi:10.15766/mep_2374-8265.11397)
Supplement: Supplementary file 1 — Preparation Resources.pptxReadiness Assurance Test.docxRAT Question Appeal Form.docxApplication Exercises.docxFacilitator Guide.docx [file mep_2374-8265.11397-s001.zip › E. Facilitator Guide.docx]

ATTENTION, STUDENTS: If you are accessing this material BEFORE it is used in your course, please do NOT read this document prior to the class session. An answer key is included in this module, which is designed to lead you through a learning experience that reinforces your knowledge of the content. Early review or dissemination of this material to others will diminish the learning opportunity and be considered academic misconduct.

**APPENDIX E. DYSLIPIDEMIA TBL FACILITATOR GUIDE**

This file contains detailed facilitation notes for Application Exercises, along with the estimated time required for all activities, including the RATs.

iRAT and tRAT are allotted 9 and 12 minutes, respectively. Note that the inclusion of more challenging RAT questions (provided in Appendix B. Readiness Assurance Test) may require additional time for the tRAT.

**Application Exercise** 1 (10 minutes with 10 minutes for discussion)

The first application exercise outlines a case vignette that prompts students to quickly identify above normal LDL-cholesterol levels, as well as additional factors that should be recognized by teams as indicative of high risk for atherosclerosis, suggesting the need to develop a patient care plan. Students will first read the patient encounter information (italicized).

***Patient Encounter***

*Gloria, a 57-year-old woman is a new patient reporting to a primary care physician for an initial visit. Her chief complaint is that she frequently experiences pain in her chest, typically after eating large meals. Her BMI is 31 and she reports smoking approximately one pack of “light” filtered cigarettes per week. Gloria reports that she leads a rather sedentary life-style as an office worker who gets little exercise.*

*Based on the initial findings, the physician reasons that Gloria is likely to be suffering from a dyslipidemia.*

| *Test* | *Result* | *Recommended Range* |
| --- | --- | --- |
| *Total Cholesterol* | *300 mg/dL* | *Desirable, less than 200 mg/dL* |
| *High-density Lipoprotein (HDL) Cholesterol* | *45 mg/dL* | *Desirable range (for age), 40-59 mg/dL* |
| *Low-density Lipoprotein (LDL) Cholesterol* | *TBD (227 mg/dL calculated)* | *Desirable, less than 130 mg/dL* |
| *Triglycerides* | *140 mg/dL* | *Desirable, less than 150 mg/dL* |

The application exercise question students are asked to discuss is:

*For patients aged 40-75 years of age, risk discussions before statin therapy initiation should include review of major risk factors such as smoking, elevated blood pressure, LDL-C, and calculated risk of atherosclerotic cardiovascular disease.*

*What else would be best for discussion with these patients?*

1. *Costs of alternative therapies such as PCSK-9*
2. *Exercise routines to reduce muscle spasms*
3. *Family history of statin use*
4. *Importance of family support for compliance in statin therapy*
5. ***Patient preferences and values in shared-decision making***

From the description of the patient encounter, student teams will note the risk factors for cardiovascular disease. The patient’s sedentary life-style, obesity, smoking behavior, and lack of exercise will be noted as common risk factors for development of dyslipidemia. The patient’s presenting complaint is chest pain and this should further concern the teams that there are already potential coronary symptoms.

At this point, the facilitator should probe teams about the level of confidence that might exist in drawing conclusions from chest pain alone. Chest pain could be the result of many conditions other than cardiovascular disease. Cardiac causes account for less than 20% of chest pain reports in primary care settings. Other causes of chest pain could be discussed as musculoskeletal, gastrointestinal, pulmonary or of psychiatric origin. The lab data in the application clearly suggest that the patient’s symptoms are likely of cardiac origin. However, students should be given pause to reach premature conclusions. For instance, many teams are able to discern the seminal importance of a risk reduction discussion with the patient, but fewer are able to maintain restraint to keep the wider possibilities in mind. Facilitators should guide a review of each option in the accompanying question to orient students to the patient’s most beneficial modifiable lifestyle factors. It is also helpful to point out to students that patients will be more receptive to advice on modifiable risk factors when faced with a need for statin therapy. After team discussions, students clearly understood that the patient’s LDL-C value indicated a dyslipidemia that would benefit from statin therapy and consideration of modifiable risks. Only rarely did student teams consider that genetic history would also require discussion, and this is a helpful lead into the following application exercises.

Facilitators will have different expectations depending on the preparation of students for the application exercises. For cases in which lab values have been presented to students in the classroom or in the pre-reading materials, the results should be self-explanatory. Risk associated with total cholesterol is very concerning. The LDL-C is a calculated value that is derived in part from the determination of triglycerides. Since the triglycerides are in the normal range, the Friedewald equation can be used [LDL-C = (Total Cholesterol) – (HDL-C) – (TGs/5)]. The facilitator should note that when triglycerides exceed 400 mg/dL, a direct LDL-C determination will be necessary as a follow-up analysis.

The emphasis in the question stem is on the best course of action and this should draw students’ attention to the one option that should be necessary in any individualized patient care exchange. Shared decision-making can be explored with students by asking, “what are considerations that need to be discussed with patient input?” Students should recognize that shared decision-making is helpful to motivate patients to consider modifiable risk factors. For this patient, smoking, lack of exercise, sedentary lifestyle and diet might have different levels of interest for the patient to begin a risk-reduction plan. Other patient preferences should be discussed in relation to potential therapies. A statin drug would be one discussion with the patient that should consider patient preferences and with special discussion of recommended treatment options, costs, family history and potential side-effects. These considerations are all helpful to discuss with the patient with respect to treatment plans, but would always be discussed with the patient’s preferences in mind.

Depending on the objectives and pre-reading assigned, facilitators can ask how this patient’s dyslipidemia ought to be categorized or coded? This question might best be presented by the facilitating physician, and should align with best practices in internal medicine that are used in an affiliated teaching hospital. For instance, the Fredrickson Classification would classify this patient’s condition as Type IIa, with elevated LDL-C and normal range triglycerides.

**Application Exercise 2** (20 minutes with 10 minutes for discussion)

In the second application exercise (below in italics), teams must interpret the supplied narrative regarding the patient’s recent diet history and submit recommendations for possible modifications of Gloria’s dietary intake pattern that would promote a more nutrient-dense, heart-healthy diet.

*1. Review Gloria’s usual dietary intake information as a team.*

*2. Describe any dietary recommendations they would suggest that could help this patient in her efforts to reduce her blood cholesterol levels and cardiovascular disease risk.*

*3. Upload their team’s suggested dietary recommendations to InteDashboard or submit in writing to a facilitator.*

***Patient Diet history (based on usual dietary intake)***

***Breakfast (home)***

*2 eggs, fried with 1 teaspoon butter*

*2 slices of bacon*

*1 small avocado*

*1 cup green tea*

***Lunch (work)***

*1 Big Mac (no bun or sauce)*

*1 large French Fries*

*1 hard-boiled egg*

*12 oz Diet Cola*

***Snack (work)***

*1 handful of mixed nuts*

*5 oz cheese*

*1 cup yogurt (whole fat)*

***Dinner (home)***

*1 large portion of spaghetti (high protein) with meat sauce/tomato sauce*

*1 low carb dinner roll with garlic butter*

*1 small mixed green salad with Italian dressing (about 2 tablespoons)*

*1 8 oz glass Diet cola*

*1 small piece of Chocolate cake*

***Snack (movie theater)***

*1 small size buttered popcorn*

*12 oz Diet Cola*

The facilitator should evaluate submissions based on the following criteria:

1. Team submissions should include dietary recommendations that meet the criteria for a patient-centered, shared decision-making, and motivational interviewing approach.

2. Submissions should include dietary pattern recommendations that meet the criteria presented in the Advance Preparation Resources (Appendix A) as evidence-based approaches for reducing blood cholesterol levels and support cardiovascular health (i.e., based on the dietary patterns that meet medical nutrition therapeutic guidelines).

3. Dietary intake recommendations should meet the elements of the behavior change model, including the 5 A’s: Assess, Advise, Agree, Assist, Arrange.

4. Submissions should include patient-centered short-term (1-2 agreed upon achievable

goals for dietary intake changes) and long-term goals (health outcome-based goals).

5. Submissions may recognize that the patient’s dietary history included a high-protein/ketogenic pattern of dietary intake, which the patient may be using as an approach to support weight loss in an effort to reduce their health risks.

Optimally, after submission of dietary pattern recommendations by all teams, submissions should be displayed in a gallery walk, followed by voting to identify the best submissions. However, factors such as large team numbers, the submission of multiple work products in this session and curricular time constraints may require the approach that we routinely take: the facilitator instead selects several teams to present their interpretations of the patient’s current diet and to discuss their rationale for their team’s recommendations. The facilitator then highlights the merits of their submitted recommendations. Following virtual TBL exercises in 2021, a facilitator provided feedback individually to each team by email after the session regarding the merits of their submissions. The motivation for this was to potentially overcome student inhibitions during virtual engagement with facilitators. This component of the exercise is optional, and we have not continued this practice since returning to in-person instruction.

Satisfactory recommendations submitted by the students should align with a low-fat dietary intake pattern geared toward lowering high cholesterol or a low sodium diet to reduce high blood pressure. Teams should recognize that the dietary intake reported in the diet history as a dietary pattern aligns well with a low carbohydrate eating plan. Superior teams should also recognize that this patient’s current dietary intake pattern resembles to some degree a “keto-like diet”. Because this is a common dietary approach for many patients attempting weight loss, it is important that students recognize and gain an understanding of this dietary approach and its impact on a patient’s health risks. As presented at the 2023 American College of Cardiology annual meeting, this diet has recently been associated with negative effects, including higher levels of LDL-cholesterol. The student teams should center on recommendations for the patient to increase fruit and vegetable intake, which are key components of the Therapeutic Lifestyle Change (TLC), the Mediterranean Diet, and the Dietary Approaches to Stop Hypertension (DASH) dietary patterns. We note that this exercise closely aligns with expected outcomes recommended by the Accreditation Council of the Graduate Medical Education for competency-based nutrition education impacting atherosclerotic cardiovascular disease prevention and treatment, as described in Table 2 of Asprey *et al*. (2018)^1^ and these competencies provided the basis for the evaluation of team dietary recommendations.

**Application Exercise 3** (20 minutes plus 10 minutes for discussion)

Students are tasked with using the patient narrative (below in italics) to generate a three-generation pedigree, determine whether an identifiable inheritance pattern exists in the constructed pedigree and if so, what dyslipidemia might be consistent with the generated pedigree.

*As described in the preparatory information, some dyslipidemias are caused by monogenic disorders, which is important information for treating not only the index patient, but potentially also the patient’s family members, while other dyslipidemias are polygenic.*

*Using the genetic family history that the physician has elicited from Gloria (below) and the attached chart of commonly-used pedigree symbols,*

1. *Work with your TBL team to draw a pedigree that most accurately reflects the family history obtained by the physician, and*
2. *State whether a recognizable pattern of inheritance exists within Gloria’s family, and if so, what type of pattern.*
3. *Which dyslipidemia is consistent with this pattern of inheritance, if one exists?*
4. *Photograph your team’s pedigree with answers to the above questions and your team number and submit your answer to the facilitators through InteDashboard or in writing.*

*Gloria reports that her mother is 89-years-old, is in generally good health and is not overweight. Her father died many years ago at the age of 52 after suffering a heart attack. Gloria remembers that he was “real heavy and also took pills for cholesterol”. Gloria has a 55-year-old brother who is also overweight and has been taking pills for “bad cholesterol” for at least five years.*

*Upon further inquiry, the physician learns that Gloria’s dad had three brothers. One died in a car accident at the age of 35, one died from a heart attack at “about 50-years-old”, and her remaining uncle is 67-years-old. Gloria describes him as about 5’10” and 240 lbs. The brothers’ mom, Gloria’s grandmother, died many years ago at age 45 due to a “stroke”.*

The image below (author owned) is an idealized pedigree consistent with the patient’s narrative of her family history. An abbreviated table of recommended pedigree symbols^2^ is supplied to each team in addition to family history narrative; while widely used, there is currently no internationally recognized body that has standardized pedigree symbol usage. It should also be emphasized that creating a family pedigree is an essential component of medical practice.


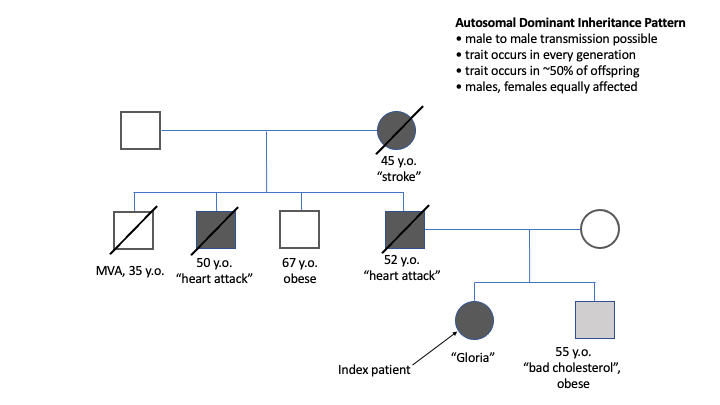


(Author owned image)

Conditions present in individuals in the family that are potentially relevant to cardiovascular disease are noted in the pedigree for clarity, along with the individual’s age, if provided. Dark grey shading is indicative of phenotypes of high concern for dyslipidemia due to the nature of the condition and the age at which an individual suffered an acute cardiovascular-related event. The lighter grey coloring of symbol for the brother of the index patient is intended to capture the ambiguity of this individual’s current disease state. No LDL-C data were provided for this individual and thus, the severity of his dyslipidemia is unknown and may be inconsistent with that observed in individuals with familial hypercholesterolemia. Also included in the image are a list of characteristics that are typically observed in pedigrees in which an autosomal dominant disorder, such as familial hypercholesterolemia, is segregating. These characteristics include the possibility of male-to-male transmission of the trait; the presence of the trait in individuals in every generation that equally affect males and females; and lastly, the expectation that approximately 50% of the offspring of an affected parent will have the trait in each generation, although it needs to be made clear that small numbers of offspring typical in human families may skew these frequencies.

Optimally, after submission of pedigrees, the submissions should be displayed in a gallery walk, followed by voting to identify the best submissions. However, factors such as large team numbers, the submission of multiple work products in this session and curricular time constraints may require the approach that we routinely take: the facilitator instead displays a subset of team submissions and asks teams to describe how they interpreted the family health history narrative to build pedigrees and their conclusions regarding potential inheritance patterns. It is expected that teams recognize that the patient’s clinical presentation, in combination with the autosomal dominant inheritance pattern of concerning cardiovascular disorders within the patient’s family, are consistent with a preliminary diagnosis of familial hypercholesterolemia. If not already commented on by teams during presentation of example pedigrees, facilitators should also highlight strengths and weaknesses of pedigrees with respect to use of widely accepted pedigree standards. A rubric for the assessment of submitted pedigrees is provided. Following virtual TBL exercises in 2021, a facilitator provided feedback individually to each team by email after the session regarding the merits of their submissions. The motivation for this was to potentially overcome student inhibitions during virtual engagement with facilitators. This component of the exercise is optional, and we have not continued this practice since returning to in-person instruction.

| **Pedigree Criteria** | **Evaluation** (exceeds, meets, or below expectations) | **Areas for Improvement** |
| --- | --- | --- |
| Organization  *[generational (parent-child and sibling) relationships, biological unions are correctly distinguished)* |  |  |
| Symbol usage and shading *(standard symbols are used to indicate sex, and appropriate symbol shading is used to indicate the presence/absence of phenotypes)* |  |  |
| Symbol notations  *[includes brief notations such as relevant phenotypes (e.g., “stroke”, “heart attack”), deceased family members and cause and age of death]* |  |  |
| Family history narrative interpretation  (submitted pedigree is a reasoned reflection of patient narrative) |  |  |
| Interpretation  *(team recognizes an autosomal dominant pattern of inheritance and provides supporting evidence)* |  |  |

In our experience, the most significant deficiencies, when present, included the absence of pedigree symbol shading to indicate affected individuals (e.g., those with a cardiovascular disease phenotype), and more rarely, incorrect usage of partial symbol shading to indicate suspected heterozygosity, which is genotypic rather than phenotypic information. Another significant but infrequent deficiency was a team’s failure to state specifically whether a recognizable inheritance pattern existed.

At this stage, facilitators should initiate discussions with students about recommendations to the index patient with regard to genetic testing. While we currently do not involve a medical ethicist in our facilitation team, the exercise could be further expanded to include a discussion of the possible need for disclosure of the potential diagnosis (or confirmed diagnosis following genetic testing) of an autosomal dominant disorder to first degree relatives (siblings, offspring, and parents) of the index patient.

**References**

1. Aspry KE, Van Horn L, Carson JAS, et al. Medical Nutrition Education, Training, and Competencies to Advance Guideline-Based Diet Counseling by Physicians: A Science Advisory From the American Heart Association. *Circulation*. Jun 5 2018;137(23):e821-e841. doi:10.1161/CIR.0000000000000563

2. Bennett RL, French KS, Resta RG, Doyle DL. Standardized human pedigree nomenclature: update and assessment of the recommendations of the National Society of Genetic Counselors. *J Genet Couns*. Oct 2008;17(5):424-33. doi:10.1007/s10897-008-9169-9
